# Inhibition of LDHB triggers DNA damage and increases cisplatin sensitivity in pleural mesothelioma

**DOI:** 10.1038/s41389-025-00571-4

**Published:** 2025-08-11

**Authors:** Yantang Lin, Christelle Dubey, Tereza Losmanova, Samuel Oevgü Yasmin, Jean-Louis Reymond, Ren-Wang Peng, Haibin Deng, Patrick Dorn, Thomas Michael Marti

**Affiliations:** 1 Department of General Thoracic Surgery, Inselspital, Bern University Hospital, University of Bern, Bern, Switzerland; 2 Oncology-Thoracic Malignancies, Department for BioMedical Research, University of Bern, Bern, Switzerland; 3 Graduate School of Cellular and Biomedical Sciences, University of Bern, Bern, Switzerland; 4 Institute of Tissue Medicine and Pathology, ITMP, University of Bern, Bern, Switzerland; 5 Department of Chemistry and Biochemistry, University of Bern, Bern, Switzerland

**Keywords:** Chemotherapy, DNA damage and repair

## Abstract

Pleural mesothelioma (PM) is an aggressive, asbestos-linked cancer with limited treatment options and a poor prognosis. Lactate dehydrogenase B (LDHB) converts lactate to pyruvate, and its silencing reduces mitochondrial metabolism, particularly nucleotide synthesis. However, whether and a role of LDHB in PM is unclear. This study aimed to investigate the effects of silencing LDHB in PM cells and their response to chemotherapy. LDHB was silenced using siRNA transfection and inducible shRNA constructs. Proliferation, colony formation, and cell viability were assessed, while DNA damage was analyzed through ɣH2AX levels. Compared to normal mesothelial cells, LDHB was highly expressed in PM cell lines. LDHB inhibition significantly reduced proliferation, cell viability, and colony formation, indicating its crucial role in PM cells. Additionally, LDHB silencing significantly increased nuclear DNA damage accumulation as indicated by elevated ɣH2AX levels, which was reversed by nucleotide supplementation. In vivo, LDHB inhibition reduced tumor growth and enhanced cisplatin’s therapeutic efficacy. LDHB silencing increased ɣH2AX levels, which were further elevated with cisplatin treatment. Our results highlight LDHB as a novel therapeutic target in PM, where its inhibition induces DNA damage and improves the efficacy of cisplatin therapy.

## Introduction

Pleural mesothelioma (PM) is a highly invasive disease with a poor prognosis that is difficult to diagnose at an early stage. If left untreated, the median survival time of patients is only 6–9 months [1]. Current treatment options for PM include surgery, chemotherapy, radiotherapy, immunotherapy, and combinations thereof, although the effectiveness of treatment is reduced by the development of resistance [2]. Recently, treatment regimens including immunotherapy have shown promise in improving survival for PM patients. However, for patients ineligible for this treatment, novel therapies have been developed but have not achieved significant clinical translation. Thus, urgent efforts are needed to develop effective treatment options for this subset of PM patients.

L-Lactate dehydrogenase (LDH) is a tetramer consisting of the two subunits LDHA and LDHB and converts lactate and NAD+ to pyruvate and NADH, respectively. LDHB favors the oxidation of lactate to pyruvate and has been reported to play an essential role in the progression of tumors from different entities [3–6], but its control during cancer progression remains poorly understood [7]. There is increasing evidence that lactate serves as the primary carbon source for mitochondrial oxidative phosphorylation (OXPHOS) and ATP production [8]. Indeed, we have previously shown that silencing LDHB expression reduced tumorigenesis and tumor growth in lung cancer. This effect was associated with decreased mitochondrial metabolism, particularly nucleotide metabolism [6].

In the clinical setting, the combination of cisplatin and pemetrexed (MTA) is still one of the gold standard chemotherapy regimens also for PM, but its efficacy is limited [9]. MTA blocks nucleotide synthesis, which is not only required for efficient DNA replication but also for the repair of DNA adducts such as the DNA intra- and interstrand crosslinks induced by cisplatin [10]. Intriguingly, our previous study revealed that, on the molecular level, LDHB silencing diminishes mitochondrial metabolism, particularly nucleotide metabolism, i.e., purine and pyrimidine metabolism [6]. Thus, we hypothesized that the nucleotide depletion induced by LDHB silencing sensitizes cancer cells to cisplatin treatment. In summary, in this study, we aimed to investigate whether LDHB silencing can inhibit tumor growth in PM per se and further augment the anticancer activity of cisplatin treatment.

## Methods

### Cell culture and reagents

PM cells (MSTO-211H, MESO4, H2052) were purchased from American Type Culture Collection (Manassas, VA, USA) and cultured in RPMI1640 medium (Cat. #8758; Sigma-Aldrich) with 10% FBS (Cat. #10270-106; Life Technologies) and 1% penicillin/streptomycin solution (Cat. #P0781, Sigma-Aldrich) at 37 °C in a humidified 5% CO_2_ incubator. All the cells were authenticated by DNA fingerprinting using highly polymorphic short tandem repeat (STR) analysis (Microsynth) and confirmed free from mycoplasma contamination. Cells with 3–15 passages were used in this study. For doxycycline treatment (Cat. #HY-N0565B; MedChem Express), a range of concentrations (0, 0.001, 0.01, 0.05, 0.1, and 0.5 μg/mL) was initially tested to determine the minimum dose required to achieve robust and reproducible protein silencing with minimal cytotoxicity. To determine the appropriate concentration for combination with cisplatin (Cat. #44033792; Sandoz) and pemetrexed (MTA) (Cat. #CS-1298; Chemscene), initial-dose response assays were performed for each drug individually in the relevant PM cell lines. Cells were treated with increasing concentrations of cisplatin (0–3 μM) and pemetrexed (0–20 μM) for 72 h and 120 h, respectively, and cell viability was assessed using the APH assay to determine the half-maximal inhibitory concentration (IC50) for each agent. Based on these results, specific concentrations were selected for subsequent combination experiments to evaluate potential synergistic effects. For adenine (Cat. #A8626; Sigma-Aldrich), guanine (Cat. #G11950; Sigma-Aldrich), cytidine (Cat. #C4654; Sigma-Aldrich), uridine (Cat. #U6381; Sigma-Aldrich), a concentration range (1.0, 3.0, 8.0, 10, 25, 100 μM) was tested to identify the effective dose that maximally rescued cytotoxicity induced by LDHB silencing. Accordingly, the optimal concentrations of each tested drug were selected based on the preliminary results and are specified in the figure legends for each experiment.

### Western blot analysis

Cell lysates were extracted in 1× RIPA Lysis and Extraction Buffer (Cat. #R0278; Sigma-Aldrich) with 1× Protease and Phosphatase Inhibitor Cocktail (Cat. #78444; Thermo Fisher Scientific) for 20 min on ice. The lysate was purified by centrifugation at 14,000× *g* for 25 min at 4 °C. Protein concentration was quantified using the BCA Protein Assay Kit (Cat. #23227; Thermo Fisher Scientific). Samples were resolved by SDS–PAGE and then transferred using Trans-Blot® Turbo™ Mini Nitrocellulose Transfer Packs (Cat. #1704158; Bio-Rad). Before staining with antibodies, membranes were blocked with Blocking Buffer (TBS) (Cat. #927-60001; LI-COR Biosciences) for 1 h at room temperature. Subsequently, the membranes were incubated with the primary antibodies of Human Lactate Dehydrogenase B/LDHB Antibody (1:20,000; Cat. #MAB9205-100; R&D Systems), Phospho-Histone H2A.X (Ser139) (20E3) Rabbit mAb (1:5000; Cat. #9718S; Cell Signaling Technology), Lactate Dehydrogenase A/LDHA Antibody (1:1000; Cat. NBP1-47822; NovusBiologicals) and beta-Actin (8H10D10) Mouse mAb (1:10,000; Cat. #3700S; Cell Signaling Technology) overnight on an orbital shaker (25 rpm) at 4 °C. After washing three times with TBS wash buffer (Tris Buffered Saline (1 tablet/500 ml; Cat. #94158-10TAB; Sigma-Aldrich Chemie GmbH) + 0.2% TWEEN 20 (Cat. #P1379; Sigma-Aldrich)), the membranes were incubated with secondary antibodies of Donkey Anti-Mouse IgG Antibody, IRDye® 680RD Conjugated (1:5000; Cat# 926-68072; LI-COR Biosciences), and IRDye 800CW Donkey anti-rabbit IgG (H + L) (1:5000; Cat# 926-32213; LI-COR Biosciences) were incubated for 30 min at room temperature. Images were acquired and analyzed using the Odyssey Infrared Imaging System (Li-COR Biosciences).

### Cell viability assay

PM cells were seeded in 96-well (2 × 10^5^) plates. For inducible cells, 24 h after seeding, cells were treated with doxycycline or doxycycline and adenine (Cat. #A8626; Sigma-Aldrich), guanine (Cat. #G11950; Sigma-Aldrich), cytidine (Cat. #C4654; Sigma-Aldrich), uridine (Cat. #U6391; Sigma-Aldrich) for 72–120 h in 100 µL cell culture medium, Cell viability was determined by PrestoBlue^TM^ Cell Viability Reagent (Cat. #A13261; Thermo Fisher Scientific) according to the manufacture’s protocol or Acid Phosphatase (APH) Assay as described [11].

### 2D colony formation assay

Cells were seeded in six-well plates (500–1000 cells/well), 24 h after, cells were cultured in either (1) cell culture medium; (2) medium with doxycycline; (3) medium with doxycycline and cisplatin; (4) medium with doxycycline and adenine (Cat. #A8626; Sigma-Aldrich), guanine (Cat. #G11950; Sigma-Aldrich), cytidine (Cat. #C4654; Sigma-Aldrich), uridine (Cat. #U6391; Sigma-Aldrich) for 10–15 days depending on growth rate. The resulting colonies were stained with crystal violet (0.5% dissolved in 25% methanol). Images of the plates were then acquired using a Kaiser eVision executive High Frequency Illuminated Copy Stand to avoid shadows. To determine the number of colonies per well, the images were then analyzed using ImageJ software (Version 1.53t). The watershed function was applied to separate closely adjacent colonies before automated counting. Colonies containing 50 or more cells were included in the count.

### 3D sphere formation assay

Tumor spheres were cultured by seeding 100–200 cells/well using MammoCult^TM^ Human Medium (Cat. #05620; STEMCELL technologies) in six-well Ultra-low Attachment plates (Cat. #CLS3471; Corning Incorporated) as described [12]. The images are analyzed using ImageJ software (Version 1.53t). The watershed function was applied to separate closely adjacent spheres before automated counting. Only spheres with a diameter of 50 µm or more were included in the count.

### Flow cytometry

Cells were harvested as indicated above. Cells were fixed with BD Cytofix/Cytoperm Fixation and Permeabilization Solution (Cat. #554722; BD Bioscience) for 15 min and permeabilized with 0.1% Triton X-100 (Cat. #X100; Sigma-Aldrich) for 10 min at room temperature. Then, cells were incubated in 100 μL PBS containing 2% FBS and 20% Fc Receptor Binding Inhibitor Functional Grade Monoclonal Antibody (Cat. #14-9161-73; Thermo Fisher Scientific) for 20 min on ice. Subsequently, cells were stained with AF-647 anti-H2A.X Phospho (Ser139) Antibody (Cat. #613408; BioLegend) overnight on a rotating wheel (3 rpm) at 4 °C and protected from light. Finally, cells were washed two times with 2% FBS and resuspended in 2% FBS containing 0.5 g/mL DAPI (Cat. #D9542; Sigma-Aldrich). All samples were measured on a BD Bioscience LSR2 upgraded flow cytometer, and 10,000 events were recorded. FlowJo V10 (Tree Star, Inc., Ashland, OR, USA, FlowJo, RRID: SCR_008520) was used to analyze FCS files.

### Cellular reactive oxygen species (ROS) detection

Cellular reactive oxygen species detection was carried out using the ROS Assay Kit from Abcam (Cat. # ab186029; Abcam) according to the manufacturer’s protocol.

### Oxygen consumption rate (OCR)

Cells were seeded overnight in Seahorse XF96 V3 PS cell culture microplates (Cat. #101085-004; Agilent Technologies) and reached 80–90% confluence on the day of the experiment. Experiments were carried out according to the manufacturer’s protocol. 1 μM oligomycin, 1.0 μM FCCP, a mixture of 1 μM rotenone and 1 μM antimycin A were added successively. The data were analyzed with Seahorse Wave (Agilent Technologies). All raw data is normalized to 50 ng DNA, which is quantified by CyQUANTTM Cell Proliferation Assay kit (Cat. # C7026; Thermo Fisher Scientific) according to the manufacturer’s protocol.

### Cell cycle analysis

Cells treated with vehicle or drugs were fixed and permeabilized with Cytofix/Cytoperm solution (Cat. # 554714, BD Biosciences). Cell cycle experiments were carried out using the Click-iT EdU Flow Cytometry Assay Kit from Invitrogen (Cat. # C10634; Thermo Scientific) according to the manufacturer’s protocol.

### Immunofluorescence microscopy

Cells grew on four-well chamber slides (Cat. #154526PK; Thermo Scientific Nunc) and reached 50–80% confluency, then fixed with 4% paraformaldehyde for 20 min at room temperature and permeabilized with 0.1% Triton X-100 for 15 min. After the cells were blocked with 1% BSA (Cat. #9998S; Cell Signaling Technology) for 1 h at 37 °C, subsequently the cells were stained with the primary antibodies of Phospho-Histone H2A.X (Ser139) (20E3) Rabbit mAb (1: 500; Cat. #9718S; Cell Signaling Technology) and Lactate Dehydrogenase B (1:500; Cat. #66425-1-Ig; Proteintech) diluted in 1% BSA at 4 °C overnight. The next day, cells were washed with PBS for twice and then incubate with secondary antibodies of Goat anti-Mouse IgG (H + L) AF-546-conjugate Secondary Antibody (1:500; Cat. #A-11030; Invitrogen) and Donkey anti-Rabbit IgG (H + L) AF-488 conjugate Secondary Antibody (1:500; Cat. #A-21206; Thermo Scientific) diluted in 1% BSA for 1 h at 37 °C. Finally, cells were mounted with mount buffer containing DAPI (Cat. # P-36931; Thermo Fisher Scientific). Images were acquired by ZEISS_LSM 710 confocal microscope and processed by ImageJ (Version 1.53t).

### Immunohistochemistry

Xenograft tumors from mice were formalin-fixed, paraffin-embedded (FFPE) and stained with hematoxylin and eosin (H&E), and antibodies against Ki-67 (1:200; Cat. #M7240; DAKO Agilent), LDHB (1:40,000; Cat. #MAB9205; R&D Systems), γH2AX (1:200; Cat. #9718S; Cell Signaling Technology) using the fully automated BOND RX® staining system (Leica Biosystems) as previously described [13]. Images were acquired using Slide Center (3DHISTECH Ltd.). Quantification was processed by ImageJ (Version 1.53t).

### Gene silencing by small interfering (siRNA) and short hairpin RNAs (shRNA)

For transient knockdown, cells were cultured in six-well plates overnight until 50–70% confluence was achieved. Cells were then transfected with 10 µL Lipofectamine 2000 (Cat. #11668027; Invitrogen) or with 3.05 µL DMH13 (a gift from Jean-Louis Reymond’s lab) in 2 mL P/S-free medium for 6 h, as described before [14]. A pooled 10 nM LDHB human siRNA Oligo Duplex (Cat. #SR320835; Origene) was used to silence LDHB, and a pooled 10 nM Trilencer-27 Universal Scrambled Negative Control siRNA Duplex was used as a negative control. For inducible stable knockdown, Tet-pLKO-neo was a gift from Dmitri Wiederschain (plasmid # 21915; Addgene). The stuffer DNA was removed from pLKO-Tet-On by AgeI/EcoRI digest and replaces with oligos encoding AgeI/ EcoRI sites and shLDHB with following sequences (shLDHB#1_F5’ CCGGCGTGATTGGAAGTGGATGTAACTCGAGTTACATCCACTTCCAATCACGTTTTTG3’; shLDHB#1_R5’AATTCAAAAACGTGATTGGAAGTGGATGTAACTCGAGTTACATCCACTTCCAATCACG3’; shLDHB#2_F5’CCGGGCTTATTTCTTCAGACACCTACTCGAGTAGGTGTCTGAAGAAATAAGCTTTTTG3’; shLDHB#2_R5’AATTCAAAAAGCTTATTTCTTCAGACACCTACTCGAGTAGGTGTCTGAAGAAATAAGC3’). Lentivirus were generated by co-transfecting 293T cells with 9 μg packing plasmid (PAX2) (Cat. #12260; Addgene), 0.9 μg envelope plasmid (VSV-G) (Cat. #35616; Addgene), 9 μg pLKO-puro-shLDHB or scramble control in Opti-MEM (Cat. #11058021; ThermoFischer) to a total volume of 225 μl using Lipofectamine 2000 (Cat. #11668027; Invitrogen). Cells were incubated for 18 h, and the growth media were replaced, and lentivirus-containing supernatant was collected 24 and 48 h later. Target cells were transduced with 8 μg/mL polybrene for 72 h and then selected with 1–2 μg/mL puromycin (Cat. # P8833-25MG; Sigma-Aldrich) for 72 h. Lentiviral expression was induced with doxycycline (0.05–0.5 µg/mL) (Cat. #HY-N0565B; MedChem Express).

### Public databases (TCGA and GEO)

LDHB analysis was performed by using BESTtools in Hiplot Pro (https://hiplot.com.cn/), a comprehensive web service for biomedical data analysis and visualization.

### In vivo experiments

Mouse studies were conducted in accordance with animal welfare guidelines and Institutional Animal Care and Ethical Committee-approved protocols. Experiments were performed in 8-week-old NSG (NOD-scid IL2Rγnull) mice, with sample size not predetermined by statistical method but rather based on preliminary experiments. In total, 1 × 10^6^ MSTO-211H cells expressing ishCTR, ishLDHB#1 or ishLDHB#2 were suspended in 100 μl PBS and growth factor-reduced basement membrane matrix (1:1) (Cat. #356231; Corning) were injected subcutaneously (left and right flank) into NSG mice. After tumors reached 50–100 mm^3^ in size, the animals from each cell line were randomly split into three groups and fed with drinking water containing: (1) 5% sucrose; (2) 5% sucrose+1 mg/mL doxycycline (Cat. #HY-N0565B; MedChem Express) or (3) 5% sucrose+1 mg/mL doxycycline and cisplatin (3 mg/kg, i.p., once a week for twice) (Cat. #44033792; Sandoz). Group sizes were determined based on prior studies to ensure sufficient statistical power to detect biologically relevant differences with an alpha level of 0.05. For (1) CTR-dox, four mice were included; (2) ishLDHB #1/#2 -dox, five mice were included; (3) CTR+dox, four mice was included; (4) ishLDHB #1/#2 +dox, five mice were included; (5) CTR+dox+cisplatin, five mice was included; (6) ishLDHB #1/#2 +dox+cisplatin, five mice was included. Mice's weight and tumor size were measured twice per week. Tumor volume was calculated as follows: (length × width × width)/2.

### Statistical analysis

Statistical analysis was performed using GraphPad Prism 9. Results were collected from at least three independent replicates for in vitro or eight tumors in vivo experiments. Error bars represent mean ± standard deviation (SD). Comparison of mean values was conducted with two-tailed unpaired Student’s *t* tests as indicated in the figure legends. In all analyses, *P* values < 0.05 were considered significant, and the significance level is reported as follows: *P < 0.05, **P < 0.01, ***P < 0.001, ****P < 0.0001.

## Results

### LDHB is required for the proliferation and survival of PM cells

**Fig. 1:**
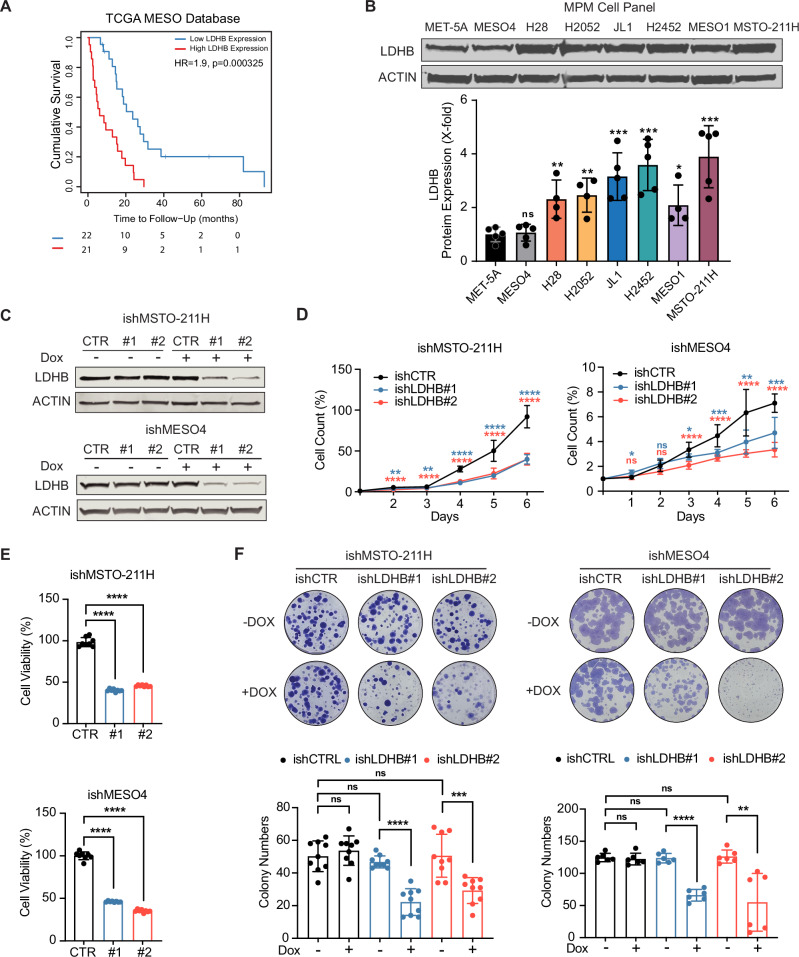
LDHB is required for the proliferation and survival of PM cells. **A** Kaplan–Meier analysis of TCGA cohort of PM patients (*n* = 86). Patients were stratified into high and low LDHB expression groups based on the median expression level. Overall survival was analyzed using the Kaplan–Meier method, and statistical significance was determined by the log-rank test. The number of patients at risk is indicated below each time point. **B** Western blot analysis of LDHB expression in the normal mesothelial cell line MET-5A and a panel of PM cell lines (MESO4, H28, H2052, JL-1, H2452, MESO-1, and MSTO-211H). β-actin (ACTIN) was used as a loading control (*n* = 3). **C** Western blot detection of LDHB expression in MSTO-211H and MESO4 cells expressed with inducible shLDHB (ishLDHB#1, ishLDHB#2) and matched shCTR (ishCTR). 0.5 µg/mL and 0.05 µg/mL doxycycline were used to induce the knockdown effect, respectively. β-actin (ACTIN) was used as a loading control (*n* = 3). **D** Proliferation analysis by cell count (*n* = 3). Doxycycline was administered 24 h after cell seeding. **E** Assessment of cell viability by acid phosphatase (APH) assay in ishMSTO-211H and ishMESO4 cells treated with 0.5 µg/mL and 0.05 µg/mL doxycycline for 72 and 120 h, respectively, normalized to ishCTR (*n* > 3). **F** Colony formation assay of ishMSTO-211H and ishMESO4 cells treated for 7–15 days with either normal culture medium or medium supplemented with 0.5 µg/mL and 0.05 µg/mL doxycycline, respectively (*n* = 3). All data represent means ± SD. *P < 0.05, **P < 0.01, ***P < 0.001, ****P < 0.0001; ns not significant; by Student’s *t* test, unpaired.

High LDHB mRNA expression correlated with poor overall survival in the TCGA PM patient cohort (*n* = 87) (Fig. 1A). Compared to the immortalized, non-malignant human mesothelial cell line cell line MET-5A, all the tested PM cell lines except for MESO4 featured significantly higher protein expression of LDHB (Fig. 1B). The siRNA-based silencing of LDHB significantly reduced LDHB protein expression (Fig. S1A), resulting in decreased formation of colonies and tumor spheres, proliferation and cell viability in three PM cell lines with different LDHB basal protein levels, i.e., MSTO-211H, MESO4 and H2052 (Fig. S1B–E). To avoid transfection-related nonspecific effects [15, 16] and enable LDHB silencing in subsequent in vivo studies, we established inducible LDHB silencing in MSTO-211H and MESO4 cells using a TET-on small hairpin RNA (ishLDHB, two different targeting sequences) or a scrambled control (ishCTR), employing a previously described viral delivery system [17]. Doxycycline (DOX) treatment resulted in a dose-dependent reduction of LDHB expression, with 0.5 μg/mL and 0.05 μg/mL DOX significantly reducing LDHB protein expression in MSTO-211H and MESO4 cells (Fig. 1C, and Figs. S1F and S2A). Interestingly, inducible LDHB silencing did not change LDHA expression levels (Fig. S2B). Consistent with the results obtained by siRNA transfection (Fig. S1), DOX-induced LDHB silencing significantly decreased proliferation, cell viability, and colony formation of MSTO-211H-ishLDHB and MESO4-ishLDHB cells compared to DOX-induced control shRNAs (Fig. 1D–F).

### LDHB silencing induces DNA damage in PM cells

**Fig. 2:**
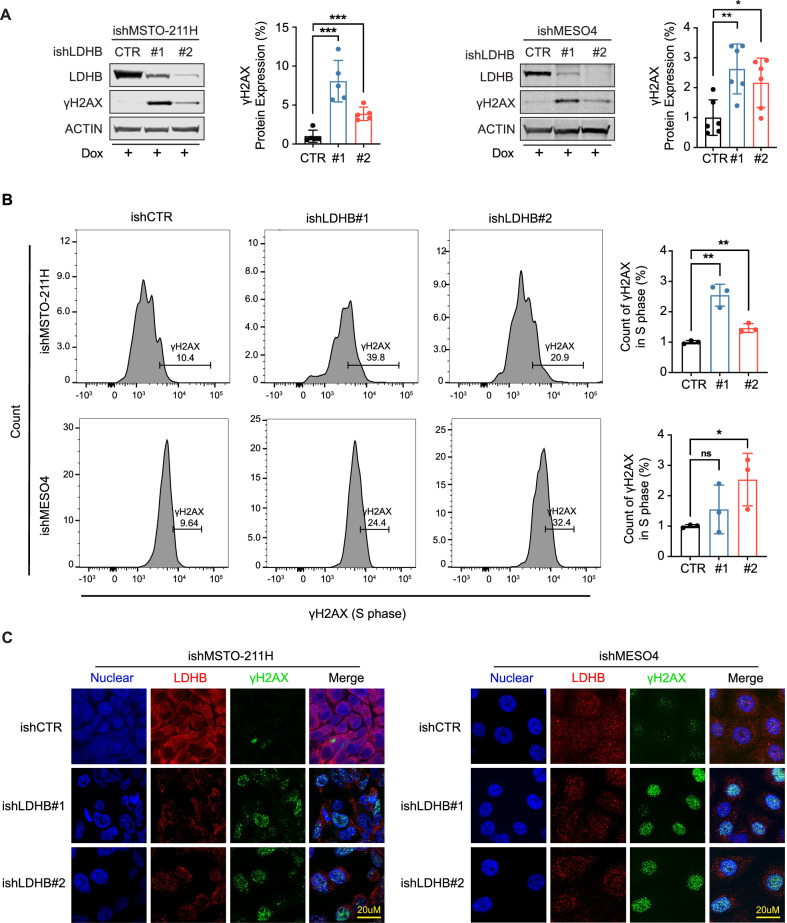
LDHB silencing induces DNA damage in PM cells. **A** Western blot analysis of LDHB and γH2AX expression in ishMSTO-211H and ishMESO4 cells. Cells were treated with 0.5 µg/mL and 0.05 µg/mL doxycycline for 72 and 120 h, respectively. Expression levels were normalized to β-actin (ACTIN) (*n* = 3). **B** Flow cytometry analysis of γH2AX in S phase of ishMSTO-211H and ishMESO4 cells. Cells were treated with 0.5 µg/mL and 0.05 µg/mL doxycycline for 72 and 120 h to induce shLDHB effect, respectively; the quantification was normalized to ishCTR (*n* = 3). **C** γH2AX foci and LDHB were assessed by immunofluorescence in ishMSTO-211H and ishMESO4 cells. Cells were treated with 0.5 µg/mL and 0.05 µg/mL doxycycline for 72 and 120 h to induce shLDHB effect, respectively (*n* = 3). All data represent means ± SD. *P < 0.05, **P < 0.01, ***P < 0.001, ****P < 0.0001; ns not significant; by Student’s *t* test, unpaired.

Next, we investigated the molecular mechanisms driving the observed phenotypic changes induced by LDHB silencing (Fig. 1). Our previous publication revealed that LDHB silencing dramatically reduces nucleotide levels, leading to mitochondrial DNA damage [6]. Given the essential role of balanced nucleotide pools in DNA replication, we investigated whether LDHB silencing also induces nuclear DNA damage. Nucleotide depletion can cause DNA replication fork stalling and, if unresolved, fork collapse, leading to DNA breaks and activation of the DNA damage response machinery [18, 19]. Indeed, inducible LDHB silencing increased the levels of γH2AX, a well-known DNA damage marker [20], in both MSTO-211H and MESO4 cells in a time-dependent manner, reaching maximal γH2AX levels 3 days and 5 days after DOX addition (Fig. 2A and Fig. S2C), respectively (see Fig. S2D for other time points). We published before that cells in S-phase of the cell cycle feature the highest H2AX phosphorylation levels [20]. Indeed, H2AX phosphorylation levels were highest in S-phase cells after LDHB silencing in both the MSTO-211H and the MESO4 cell lines (Fig. 2B and Figs. S3–4). DNA replication stress results in pan-nuclear γH2AX staining, whereas DNA double-strand breaks lead to the formation of distinct nuclear foci [20]. Indeed, a notable increase in γH2AX foci was detectable in both cell lines after LDHB inhibition (Fig. 2C), suggesting that LDHB silencing results in the induction of DNA double-strand breaks. We described previously that the accumulation of DNA damage is associated with increased induction of apoptosis [21, 22]. However, LDHB inhibition did not result in a significant induction of apoptosis at the tested time points (Figs. S5 and 6). Further, LDHB inhibition did not significantly change the cell cycle distribution in either of the tested PM cell lines (Fig. S7). To determine the fraction of proliferating cells, we quantified the incorporation of the thymidine nucleoside analog 5-ethynyl-2’-deoxyuridine (EdU), a marker for cells actively synthesizing DNA [23]. In the MSTO-211H cell line, EdU incorporation was indeed reduced 24 and 48 h after LDHB silencing. Unexpectedly, however, EdU incorporation was increased at 72 h after LDHB silencing (Fig. S8). Further, the MESO4 cell line showed increased EdU incorporation at all four time points analyzed (48, 72, 96, and 120 h) after LDHB silencing (Fig. S9). Thus, LDHB silencing resulted in the accumulation of DNA damage and also affected the fraction of proliferating cells.

### LDHB silencing induces DNA damage by interfering with nucleotide metabolism

**Fig. 3:**
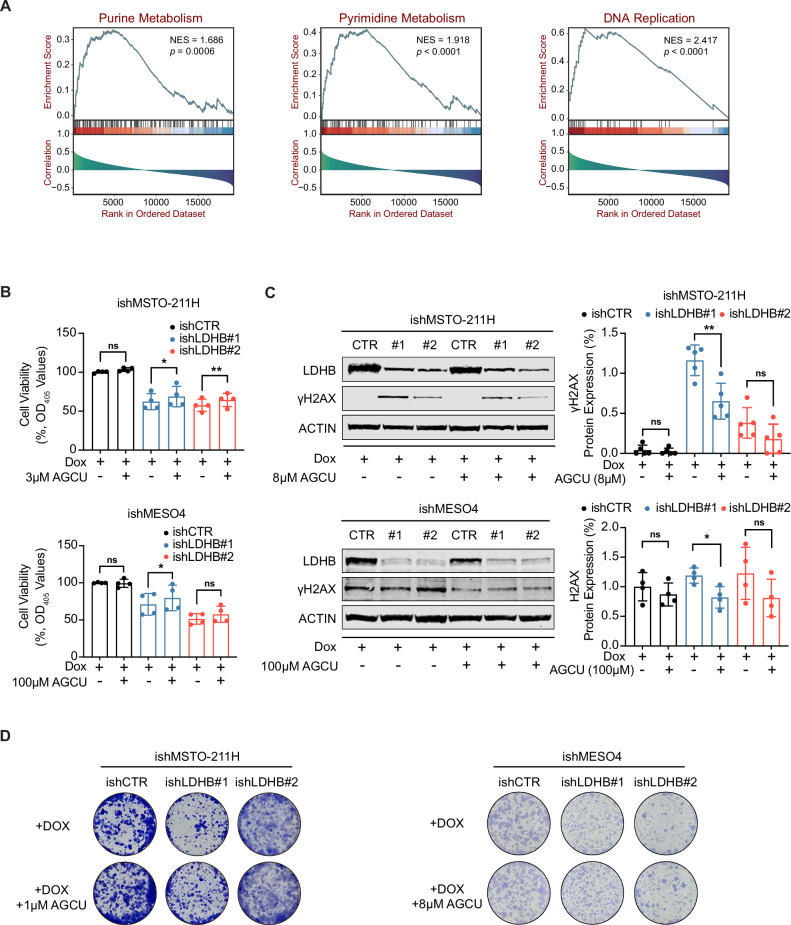
LDHB silencing induces DNA damage by interfering with nucleotide metabolism. **A** Gene Set Enrichment Analysis (GSEA) of the TCGA dataset revealed enrichment of the purine metabolism, pyrimidine metabolism, and DNA replication pathway in Kyoto Encyclopedia of Genes and Genomes (KEGG) for samples stratified by LDHB expression. **B** Cell viability assay by acid phosphatase (APH) in ishMSTO-211H and ishMESO4 cells, treated with 0.5 µg/mL and 0.05 µg/mL doxycycline, supplemented with or without 3 µM and 100 µM AGCU (adenine, guanine, cytosine, and uridine) for 72 h and 120 h, respectively, normalized to ishCTR (*n* > 3). **C** Western blot analysis of LDHB and γH2AX expression in ishMSTO-211H and ishMESO4 cells. Cells were treated with 0.5 µg/mL and 0.05 µg/mL doxycycline supplemented with or without 8 µM and 100 µM AGCU (adenine, guanine, cytosine, and uridine) for 72 h and 120 h, respectively (*n* = 3). **D** Colony formation assay in ishMSTO-211H and ishMESO4 cells. Cells were cultured in medium with 0.5 µg/mL and 0.05 µg/mL doxycycline (above panel), 1 µM and 8 µM AGCU (adenine, guanine, cytosine, and uridine) were supplemented in the medium for the bottom panel (*n* = 3). All data represent means ± SD. *P < 0.05, **P < 0.01, ***P < 0.001, ****P < 0.0001; ns not significant; by Student’s *t* test, unpaired.

Next, we aimed to elucidate the molecular mechanisms underlying the increased DNA damage accumulation upon LDHB silencing. We showed before that silencing LDHB disrupts mitochondrial metabolism [6], and others have shown that mitochondrial stress can lead to the accumulation of reactive oxygen species (ROS), a well-known inducer of DNA damage [24, 25]. Indeed, LDHB inhibition also impaired mitochondrial metabolism in the tested PM cell lines, as evidenced by a decreased oxygen consumption rate (OCR) (Fig. S10A). However, LDHB silencing did not significantly increase the average ROS levels in either of the tested PM cell lines at the tested time points (Fig. S10B). To investigate cellular pathways that may represent specific dependencies in PM, we conducted an integrated analysis of transcriptomic and clinical data from PM patients available in the Cancer Genome Atlas (TCGA) [26]. Gene set enrichment analysis (GSEA) indicated that expression of the LDHB gene was significantly associated with nucleotide metabolism and DNA damage response pathways (Fig. 3A and Fig. S11A). Indeed, upon LDHB silencing, supplementation of nucleotide precursors slightly but significantly increased cell viability in MSTO-211H cells and in MESO4 after silencing with the ishLDHB#1 construct, but not with the ishLDHB#2 construct in ishMESO4 (Fig. 3B and Fig. S11B). In addition, Western blot analysis revealed that nucleotide supplementation partially mitigated the elevated γH2AX expression levels induced by LDHB silencing (Fig. 3C and Fig. S11C). Finally, nucleotide supplementation also partially rescued the reduction in colony formation induced by LDHB silencing in the two cell lines MSTO-211H and MESO4 (Fig. 3D and Fig. S11D). In conclusion, after LDHB silencing, nucleotide supplementation partially reverses the accumulation of DNA damage, indicating that reduced nucleotide levels contribute, at least in part, to the reduction in proliferation, viability, and colony formation in PM cells.

### LDHB inhibition sensitizes PM cells to cisplatin treatment

**Fig. 4:**
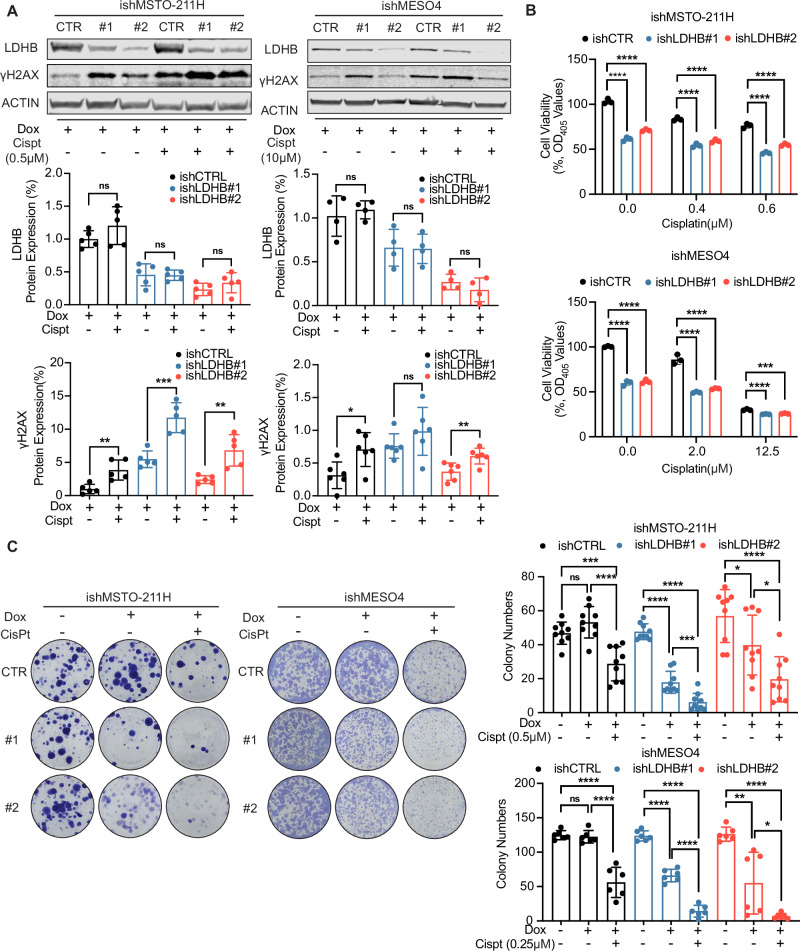
LDHB inhibition sensitizes PM cells to cisplatin treatment. **A** Western blot analysis of LDHB and γH2AX expression in ishMSTO-211H and ishMESO4. Cells were treated with doxycycline alone as described before or in combination with 0.5 µM and 10 µM cisplatin 24 h after seeding for 72 h and 120 h, quantification of the expression level was normalized to β-actin (ACTIN) (*n* = 3). **B** Cell viability assay by acid phosphatase (APH) in ishMSTO-211H and ishMESO4 cells, cells were treated with doxycycline alone as described before or in combination with cisplatin 24 h after seeding for 72 h and 120 h, normalized to non-cisplatin treated ishCTR (*n* = 3). **C** Colony formation assay in ishMSTO-211H and ishMESO4 cells. Cells were treated with normal culture medium, medium with 0.5 µg/mL and 0.05 µg/mL doxycycline alone or in combination with 0. 5 µM and 0.25 µM cisplatin, respectively, for 7–15 days (*n* = 3). All data represent means ± SD. *P < 0.05, **P < 0.01, ***P < 0.001, ****P < 0.0001; ns not significant; by Student’s *t* test, unpaired.

Subsequently, we aimed to test our hypothesis that the nucleotide depletion induced by LDHB silencing sensitizes cancer cells to cisplatin treatment. We have previously shown that pemetrexed-induced nucleotide depletion sensitizes cancer cells to the subsequent induction of DNA damage by both radiotherapy and cisplatin treatment [21, 27, 28]. Given that LDHB silencing hampers nucleotide synthesis, we sought to investigate whether LDHB inhibition could potentiate cisplatin treatment. Both after control transfection and after LDHB silencing, LDHB protein levels were not altered by cisplatin treatment. However, the combination of cisplatin treatment with LDHB inhibition further increased γH2AX expression in both MSTO-211H and MESO4 cell lines compared to either treatment alone (Fig. 4A and Fig. S12A). Indeed, higher LDHB expression is associated with increased sensitivity to cisplatin treatment in PM (Fig. S12B), which is in agreement with the more pronounced increase in H2AX phosphorylation in MSTO-211H cells featuring higher LDHB levels (Figs. 1B and 4A). Consistently, reduced viability and colony formation were observed when cisplatin treatment was combined with LDHB inhibition compared to the two single treatments (Fig. 4B, C and Fig. S12C). Interestingly, additional treatment with the nucleotide synthesis inhibitor pemetrexed (MTA) did not further reduce colony formation when combined with either LDHB inhibition alone or combined with cisplatin treatment (Fig. S12C). Our results indicate that the combination of cisplatin with LDHB inhibition leads to increased accumulation of DNA damage, thereby reducing the colony formation capacity of PM cells, suggesting a potential new avenue for PM therapy.

### Targeting LDHB reduces PM tumor growth and improves cisplatin therapy

**Fig. 5:**
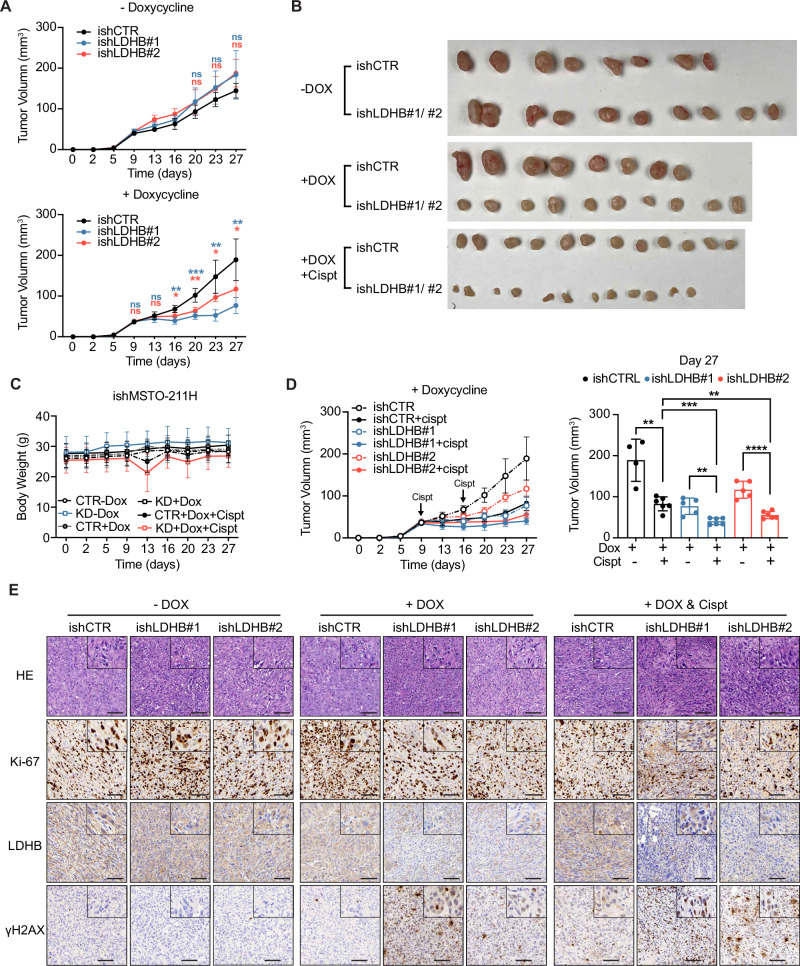
Targeting LDHB reduces PM tumor growth and improves cisplatin therapy. **A** Growth curves of ishMSTO-211H xenograft tumors with 5% sucrose in water (above panel) and in combination with 1 mg/kg doxycycline (bottom panel) over time. **B** Images of ishMSTO-211H xenograft tumors treated with 5% sucrose, 5% sucrose+1 mg/kg doxycycline, 5% sucrose+1 mg/kg doxycycline in combination with cisplatin (3 mg/kg). **C** Body weight of ishMSTO-211H xenograft tumors treated with 5% sucrose, 5% sucrose+1 mg/kg doxycycline, 5% sucrose+1 mg/kg doxycycline in combination with cisplatin (3 mg/kg). **D** Growth curves of ishMSTO-211H xenograft tumors treated with 5% sucrose, 5% sucrose+1 mg/kg doxycycline, 5% sucrose+1 mg/kg doxycycline in combination with cisplatin (3 mg/kg) for the indicated time (left panel). Quantification of the tumor volume at day 27 (right panel). **E** Representative images or H&E staining and IHC analysis for Ki-67, LDHB, and γH2AX of ishMSTO-211H xenograft tumors treated with 5% sucrose, 5% sucrose+1 mg/kg doxycycline, 5% sucrose+1 mg/kg doxycycline in combination with cisplatin (3 mg/kg). Scale bar, 100 µm. Tumor growth was monitored in six groups: CTR -dox (*n* = 4), ishLDHB #1/#2 -dox (*n* = 5), CTR +dox (*n* = 4), ishLDHB #1/#2 +dox (*n* = 5), CTR +dox+cisplatin (*n* = 5), and ishLDHB #1/#2 +dox+cisplatin (*n* = 5). All data represent means ± SD. *P < 0.05, **P < 0.01, ***P < 0.001, ****P < 0.0001; ns not significant; by Student’s *t* test, unpaired.

Next, we aimed to test the effect of LDHB silencing on tumor growth in vivo. Consistent with our in vitro findings, compared to control tumors, LDHB inhibition notably reduced tumor growth over time (Fig. 5A, B and Fig. S14) without evident side effects, i.e., the body weight of the animals did not decrease during the experiment (Fig. 5C). Cisplatin treatment reduced tumor growth over time to a similar extend than LDHB silencing (Fig. 5D). Remarkably, combining cisplatin treatment with LDHB inhibition further decreased tumor volume compared to both single treatments (Fig. 5B–D). On the molecular level, LDHB inhibition significantly impeded PM cancer cell proliferation, as indicated by a reduction of the fraction of cells, which stained positive for Ki-67, a well-established proliferation marker [29]. The fraction of Ki-67-positive cells was even further when LDHB silencing was combined with cisplatin treatment (Fig. 5E and Fig. S13). Intriguingly, cisplatin treatment and LDHB silencing both increased the levels of DNA damage, as indicated by the increased fraction of γH2AX-positive cells, and the combination therapy further augmented DNA damage accumulation compared to both single treatments (Fig. 5E and Fig. S13). In conclusion, our in vivo experiments have shown that silencing LDHB induces DNA damage in PM cancer cells, reduces tumor growth, and improves cisplatin therapy, indicating the potential of targeting LDHB for PM therapy.

## Discussion

Our study revealed that LDHB is necessary for the proliferation, survival, and colony formation of PM cells (Fig. 1). However, the role of LDHB in different cancers is complex. In several cancers, including prostate, bladder, and hepatocellular carcinoma, LDHB is silenced due to promoter methylation. In contrast, increased LDHB expression was described in lung and pancreatic adenocarcinomas, osteosarcoma, and testicular germ cell tumors (see ref. [30] for references). Similarly, silencing of LDHB reduced the growth of some cancer cell lines, whereas glycolysis was promoted in pancreatic cancer cells, resulting in increased proliferation, invasion, and migration in hypoxia [31]. Thus, it will be interesting to explore whether expression profiles linked to the tissue of origin or genetic alterations associated with cancer could serve as markers for sensitivity to LDHB silencing.

We have shown before that LDHB silencing results in the accumulation of damaged mitochondrial DNA [6], which lacks protective histones [32]. Others have shown before that silencing LDHB in auditory cells promoted age-related hearing loss and increased sensitivity to cisplatin, a known DNA-damaging agent [33]. In this study, we found that silencing of LDHB also leads to the accumulation of nuclear DNA damage as indicated by increased phosphorylation of the nuclear protein histone H2AX (Fig. 2). Thus, it will be interesting to elucidate if the accumulation of nuclear and/or mitochondrial DNA damage correlates with sensitivity to LDHB silencing.

Disruption of mitochondrial metabolism can result in ROS accumulation [25], which, when not neutralized, results in accumulation of oxidative DNA damage. Interestingly, in this study, we found that LDHB silencing reduced the OCR but this was not accompanied by a significant increase in average ROS levels in either of the two PM cell lines (Fig. S10A, B). This is in agreement with our previous study, which revealed that LDHB silencing similarly did not elevate ROS levels in lung cancer cells [34]. However, we previously demonstrated that LDHB silencing dramatically reduces GSH levels in cancer cells [6, 34]. Therefore, we speculate that ROS production might actually be decreased upon LDHB silencing. In the context of reduced GSH levels, this reduction in ROS production might result in similar steady-state ROS levels, even though the overall flux of ROS molecules might be diminished. Future studies aimed at characterizing the specific nature of the DNA damage caused by LDHB silencing may provide valuable insights into the precise molecular mechanisms underlying the observed accumulation of DNA damage.

This study revealed that nucleotide supplementation partially rescues nuclear DNA damage induced by LDHB silencing (Fig. 3). This is in agreement with our previous study, which revealed that silencing LDHB reduces mitochondrial metabolism, particularly nucleotide metabolism, i.e., purine and pyrimidine metabolism [6]. We have previously discussed that an imbalance in the nucleotide pools can lead to blocked DNA replication forks, which, if not repaired, will break down and lead to the formation of DNA double-strand breaks [18]. Interesting in this context of nucleotide metabolism is our finding that EdU incorporation after LDHB silencing is actually increased in the latest time point in MSTO-211H cell line and at all tested time points in the MESO4 cell line (Figs. S8 and 9). One possible interpretation of these data is that LDHB silencing leads to an increased rate of DNA synthesis. However, this would contrast with the reduced proliferation and viability of PM cells documented in this study (Figs. 1 and 3, respectively). An alternative explanation could be that the supplementation with EdU, a thymidine analog, actually rescues a nucleotide deficiency induced by LDHB silencing. On the molecular level, EdU is phosphorylated into EdUTP and competes with dTTP for incorporation into DNA during replication [35]. Thus, we hypothesize that the lack of dTTP leads to the formation of stalled DNA replication forks, which are then restarted upon the addition of EdU. Since EdU supplementation specifically compensates for the dTTP deficiency, while the levels of the other three nucleotides are either not restored or only restored to a lesser extent, the reduction of dTTP could represent the rate-limiting step for DNA replication after LDHB inactivation. Intriguingly, pemetrexed treatment, which inhibits Thymidylate Synthetase (TYMS) and thus thymidine synthesis, did not further reduce colony formation when combined with LDHB silencing (Fig. S12C). This suggests that the two treatments are epistatic, meaning they target the same pathway. Notably, the rate-limiting step of thymidine synthesis is catalyzed by DHODH, an enzyme located in the mitochondrial membrane [36], which is also the proposed localization site of LDHB [37]. Although it is tempting to speculate that LDHB inhibition interferes with DHODH-mediated thymidine synthesis, further experiments are needed to confirm this at the molecular level.

Notably, LDHB inhibition did not result in a significant induction of apoptosis at the tested time points (Figs. S5 and 6). As apoptosis serves as a critical barrier to tumor development, anti-apoptotic adaptations is a prominent hallmark of cancer, including PM [38, 39]. In this context, we recently demonstrated that LDHB silencing sensitizes not only NSCLC, fibrosarcoma, and pancreatic cancer cells but also MSTO-211H cells, i.e., a biphasic PM cell line, to ferroptosis induction [40]. Others have shown that cisplatin treatment mainly triggers apoptosis and necroptosis in MSTO-211H cells but to a minor extend also ferroptosis [41]. In addition, we demonstrated that LDHB silencing combined with radiotherapy induces senescence in A549 NSCLC cells [42]. We also observed senescence induction after pemetrexed-cisplatin combination therapy in PM cells [28]. Therefore, further studies are needed to determine whether apoptosis-independent cell death pathways, such as senescence or ferroptosis, contribute to the reduced proliferation, viability, and colony formation observed after combining LDHB silencing with cisplatin treatment.

Our study revealed that LDHB inhibition sensitizes PM cells to cisplatin treatment (Fig. 4). LDHB expression correlates with response to neoadjuvant chemotherapy in breast cancer [43]. It was shown before that silencing of LDHB renders oral squamous cell carcinoma cells more sensitive to Paclitaxel, which disrupts mitotic spindle disassembly and thus blocks cell division [44]. In addition, we recently showed that targeting LDHB sensitizes lung cancer cells to radiotherapy [42]. Therefore, it would be valuable to investigate whether silencing LDHB renders cancer cells from additional tumor types more sensitive to cisplatin and other DNA-damaging treatments, such as radiotherapy. Indeed, we recently demonstrated that LDHB suppresses mitochondria-associated ferroptosis, a process linked to the accumulation of lipid peroxidation in various tumor types [40]. Future studies are needed to determine the extent to which DNA damage or lipid peroxidation contributes to the observed anti-tumor effects of LDHB silencing and whether these contributions vary across different tumor types.

A limitation of our study is that we evaluated the effect of LDHB silencing, both alone and in combination with cisplatin treatment, exclusively in a subcutaneous tumor model using immunocompromised hosts (Fig. 5). However, a recent study showed that overexpression of LDHB suppressed hepatocellular carcinoma growth in immunocompetent but not in immunodeficient mice, suggesting that the host immune system was involved in the LDHB-medicated tumor suppression [45]. Therefore, further studies using orthotopic tumor models in immunocompetent hosts will be essential to understand how targeting LDHB affects tumor growth within an intact tumor immune microenvironment. A previous study reported that cisplatin treatment reduces LDHA expression in human oral squamous cell carcinoma cell lines [46], suggesting that changes in LDHA levels may add complexity to the interpretation of LDHB-mediated effects in this context. Nevertheless, this experimental approach will enable the exploration of LDHB’s potential as a therapeutic target and prognostic biomarker for cancer immunotherapy. Furthermore, we used MET-5A as a mesothelial control cell line, although it is immortalized via transfection with the SV40 early region and the Rous sarcoma virus long terminal repeat. Therefore, including primary, non-immortalized mesothelial cells from healthy donors would offer a more physiologically relevant control. Finally, in 2015, the anti-epileptic drug stiripentol was reported to inhibit both LDHA and LDHB [47]. More recently, AXKO-0046 was identified as the first highly selective inhibitor of human LDHB [48]. Our group is currently investigating the anticancer effects of various LDH inhibitors in a separate study.

### Conclusion

In summary, this study revealed that LDHB is highly expressed in PM cell lines and that silencing LDHB reduces cell proliferation, viability, and colony formation. LDHB inhibition also increased DNA damage, particularly during the S-phase, and improved the effectiveness of cisplatin treatment. Thus, targeting LDHB could be a promising strategy to enhance the effectiveness of chemotherapy-based combination therapies for PM and potentially other tumor types.

## Supplementary information

Supplementary Figures S1-14

## Data Availability

All data generated or analyzed during this study are included in this published article and supplementary information files.
