# Supplementary material for: Inhibition of LDHB triggers DNA damage and increases cisplatin sensitivity in pleural mesothelioma
Source: Oncogenesis. 2025 Aug 11;14(1):28. doi: 10.1038/s41389-025-00571-4 (PMC12340043; doi:10.1038/s41389-025-00571-4)
Supplement: Supplementary file 1 — Supplementary Figures S1-14 [file 41389_2025_571_MOESM1_ESM.pdf]

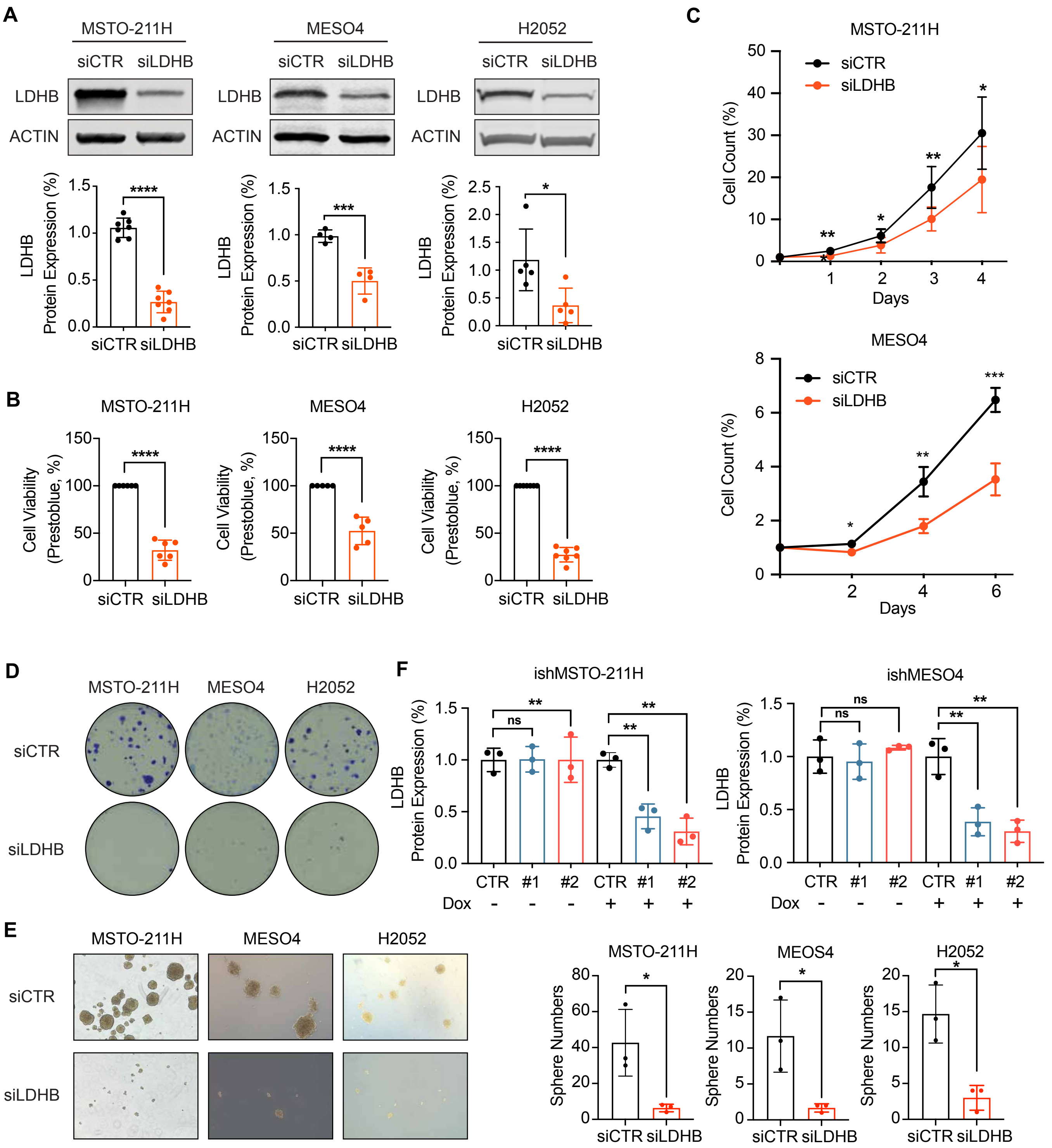

A
MSTO-211H
MESO4
H2052
siCTR siLDHB
LDHB
ACTIN
LDHB Protein Expression (%)
siCTR siLDHB
****
***
*
B
Cell Viability (Prestoblue, %)
C
Cell Count (%)
Days
D
E
Sphere Numbers
MEOS4
F
ishMSTO-211H
ishMESO4
ns
**
CTR #1 #2 CTR #1 #2
Dox - - - + + +

Figure S1. LDHB is essential for the proliferation and survival of PM cells. A. Western blot detection of LDHB expression in MSTO-211H, MESO4 and H2052 siCTR and siLDHB cells (above panel). Quantification of the western blot analysis (bottom panel) β-actin (ACTIN) was used as a loading control (n=3). B. Assessment of cell viability in MSTO-211H, MESO4 and H2052 siCTR and siLDHB cells for 72h and 120h respectively, normalized to ishCTR (n>3). C. Proliferation analysis by cell count in MSTO-211H and MESO4 siCTR and siLDHB cells (n=3). D. Colony formation assay of siCTR and siLDHB cells in MSTO-211H, MESO4 and H2052 (n=3). E. Sphere colony formation analysis assay of MSTO-211H, MESO4 and H2052 siCTR and siLDHB cells (n=3). F. Quantification of LDHB expression in MSTO-211H and MESO4 cells expressing inducible shLDHB (ishLDHB#1, ishLDHB#2) and matched shCTR (ishCTR), see figure 1C. 0.5µM and 0.05µM Doxycycline was used to induce knockdown effect respectively. β-actin (ACTIN) was used as a loading control (n=3). All data represent means ± SD. *p<0.05, **p<0.01, ***p<0.001, ****p<0.0001; ns, not significant; by Student's t test, unpaired.

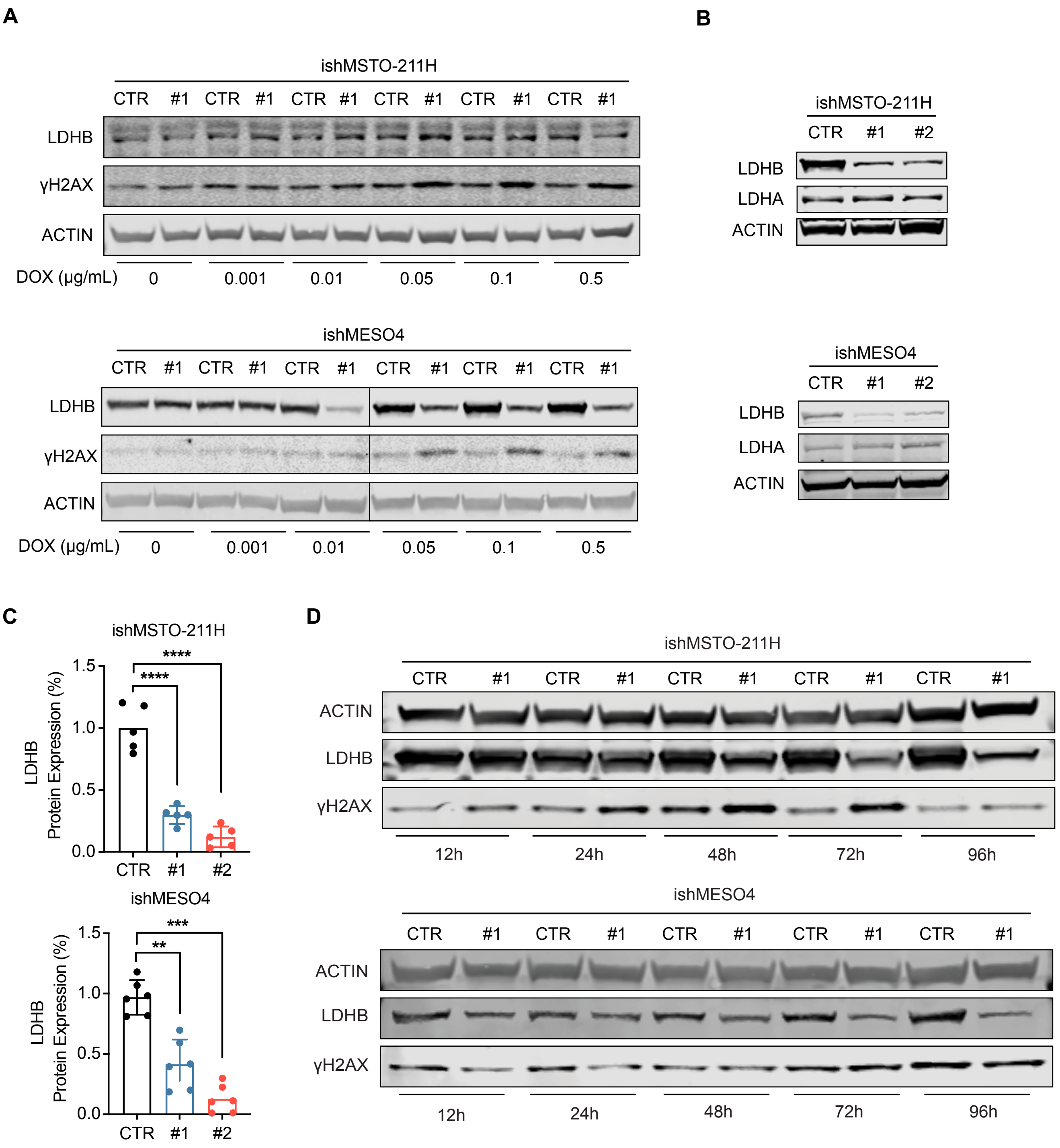
A
ishMSTO-211H
CTR #1 CTR #1 CTR #1 CTR #1 CTR #1 CTR #1
LDHB
γH2AX
ACTIN
DOX (μg/mL) 0 0.001 0.01 0.05 0.1 0.5
ishMESO4
CTR #1 CTR #1 CTR #1 CTR #1 CTR #1 CTR #1
LDHB
γH2AX
ACTIN
DOX (μg/mL) 0 0.001 0.01 0.05 0.1 0.5
B
ishMSTO-211H
CTR #1 #2
LDHB
LDHA
ACTIN
ishMESO4
CTR #1 #2
LDHB
LDHA
ACTIN
C
ishMSTO-211H
LDHB Protein Expression (%)
0.0 0.5 1.0 1.5
****
****
CTR #1 #2
ishMESO4
LDHB Protein Expression (%)
0.0 0.5 1.0 1.5
***
**
CTR #1 #2
D
ishMSTO-211H
CTR #1 CTR #1 CTR #1 CTR #1 CTR #1
ACTIN
LDHB
γH2AX
12h 24h 48h 72h 96h
ishMESO4
CTR #1 CTR #1 CTR #1 CTR #1 CTR #1
ACTIN
LDHB
γH2AX
12h 24h 48h 72h 96h

Figure S2. LDHB silencing induces DNA damage in PM cells. A. Western blot analysis of LDHB and γH2AX expression in ishMSTO-211H and ishMESO4 cells with various concentration of doxycycline for 72 and 120h to induce shLDHB effect respectively, β-actin (ACTIN) was used as a loading control (n=3). B. Western blot analysis of LDHB and LDHA expression in ishMSTO-211H and ishMESO4 cells expressed with inducible shLDHB (ishLDHB#1, ishLDHB#2) and matched shCTR (ishCTR). 0.5µg/mL and 0.05µg/mL doxycycline was used to induce knockdown effect respectively. β-actin (ACTIN) was used as a loading control (n=3). C. Quantification of LDHB expression in ishMSTO-211H and ishMESO4 cells expressing inducible shLDHB (ishLDHB#1, ishLDHB#2) and matched shCTR (ishCTR), see figure 2A. 0.5µg/mL and 0.05µg/mL doxycycline was used to induce knockdown effect respectively. β-actin (ACTIN) was used as a loading control (n=3). D. Western blot analysis of LDHB and γH2AX expression in ishMSTO-211H and ishMESO4 cells with different time points after treating with 0.5µg/mL and 0.05µg/mL doxycycline to induce shLDHB effect respectively, the quantification of the expression level was normalized to β-actin (ACTIN) (n=3). All data represent means ± SD. *$p<0.05$, **$p<0.01$, ***$p<0.001$, ****$p<0.0001$; ns, not significant; by Student's t test, unpaired.

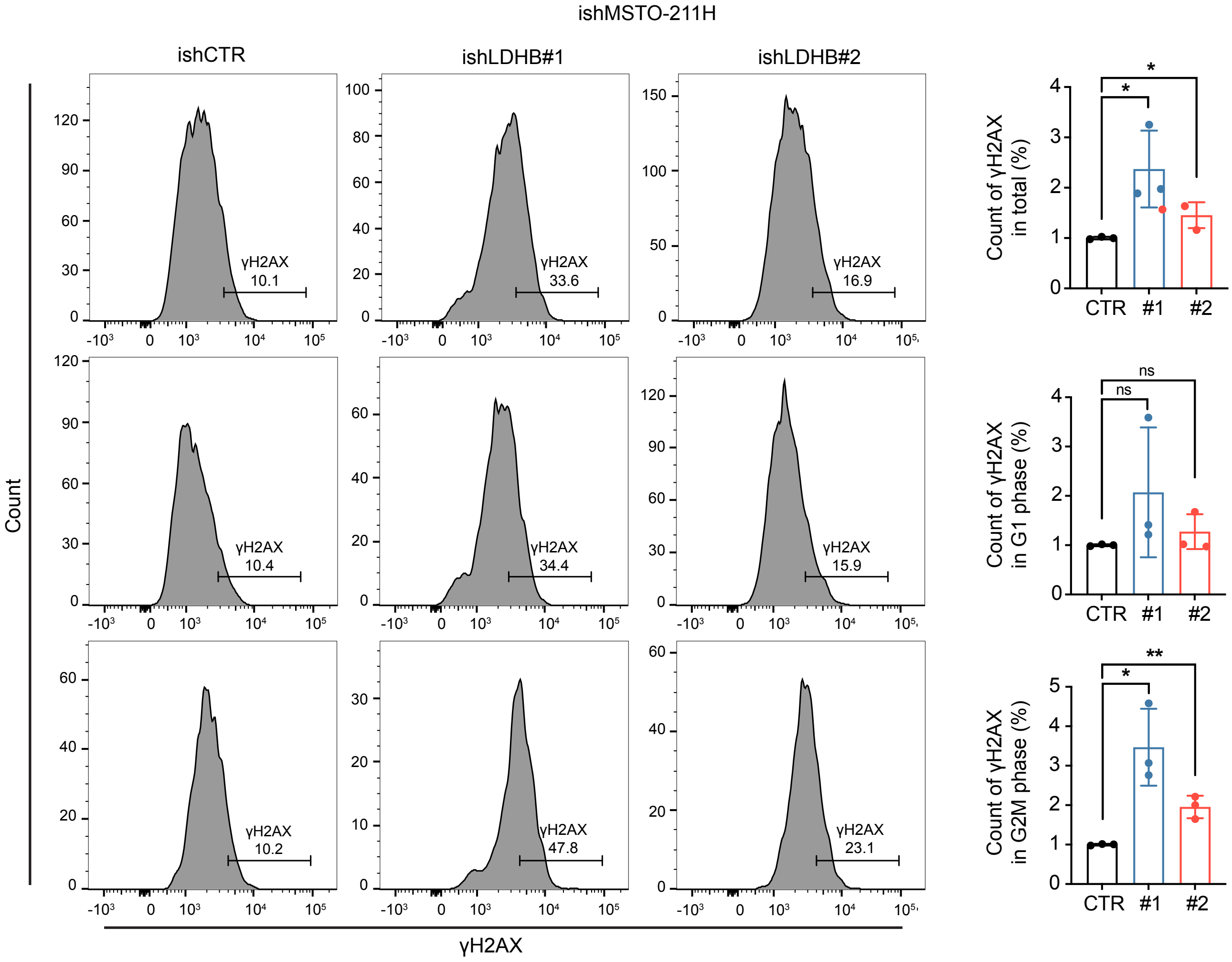

ishMSTO-211H
ishCTR
ishLDHB#1
ishLDHB#2
Count
γH2AX
γH2AX 10.1
γH2AX 33.6
γH2AX 16.9
γH2AX 10.4
γH2AX 34.4
γH2AX 15.9
γH2AX 10.2
γH2AX 47.8
γH2AX 23.1
Count of γH2AX in total (%)
Count of γH2AX in G1 phase (%)
Count of γH2AX in G2M phase (%)
CTR
#1
#2
ns

Figure S3. LDHB inhibition induces DNA damage in ishMSTO-211H cells. Flow cytometry analysis of γH2AX in G1, G2M and whole cell cycle phase of ishMSTO-211H (left panel). Quantification of γH2AX in corresponding cell cycle phase, normalized to ishCTR (n=3) (right panel). Cells were treated with 0.5µg/mL doxycycline for 72 hours to induce shLDHB effect. All data represent means ± SD. *p<0.05, **p<0.01, ***p<0.001, ****p<0.0001; ns, not significant; by Student's t test, unpaired.

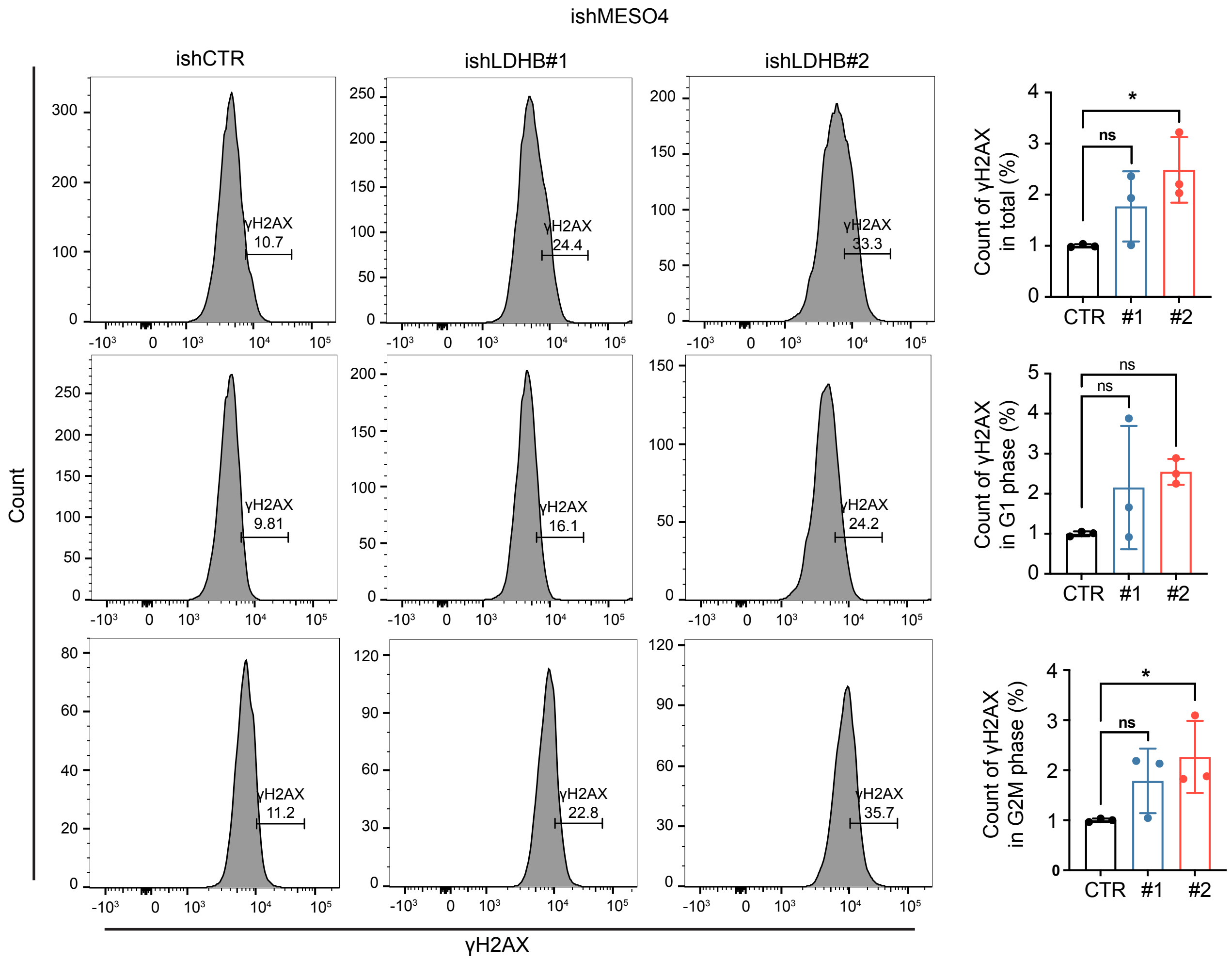

ishMESO4
ishCTR
ishLDHB#1
ishLDHB#2
Count
γH2AX
γH2AX 10.7
γH2AX 24.4
γH2AX 33.3
γH2AX 9.81
γH2AX 16.1
γH2AX 24.2
γH2AX 11.2
γH2AX 22.8
γH2AX 35.7
Count of γH2AX in total (%)
Count of γH2AX in G1 phase (%)
Count of γH2AX in G2M phase (%)
CTR
#1
#2
ns
*

Figure S4. LDHB inhibition induces DNA damage in MESO4 cells. Flow cytometry analysis of γH2AX in G1, G2M and whole cell cycle phase of ishMESO4 (left panel). Quantification of γH2AX in corresponding cell cycle phase, normalized to ishCTR (n=3) (right panel). Cells were treated with 0.05µg/mL doxycycline for 120 hours to induce shLDHB effect. All data represent means ± SD. *$p<0.05$, **$p<0.01$, ***$p<0.001$, ****$p<0.0001$; ns, not significant; by Student's t test, unpaired.

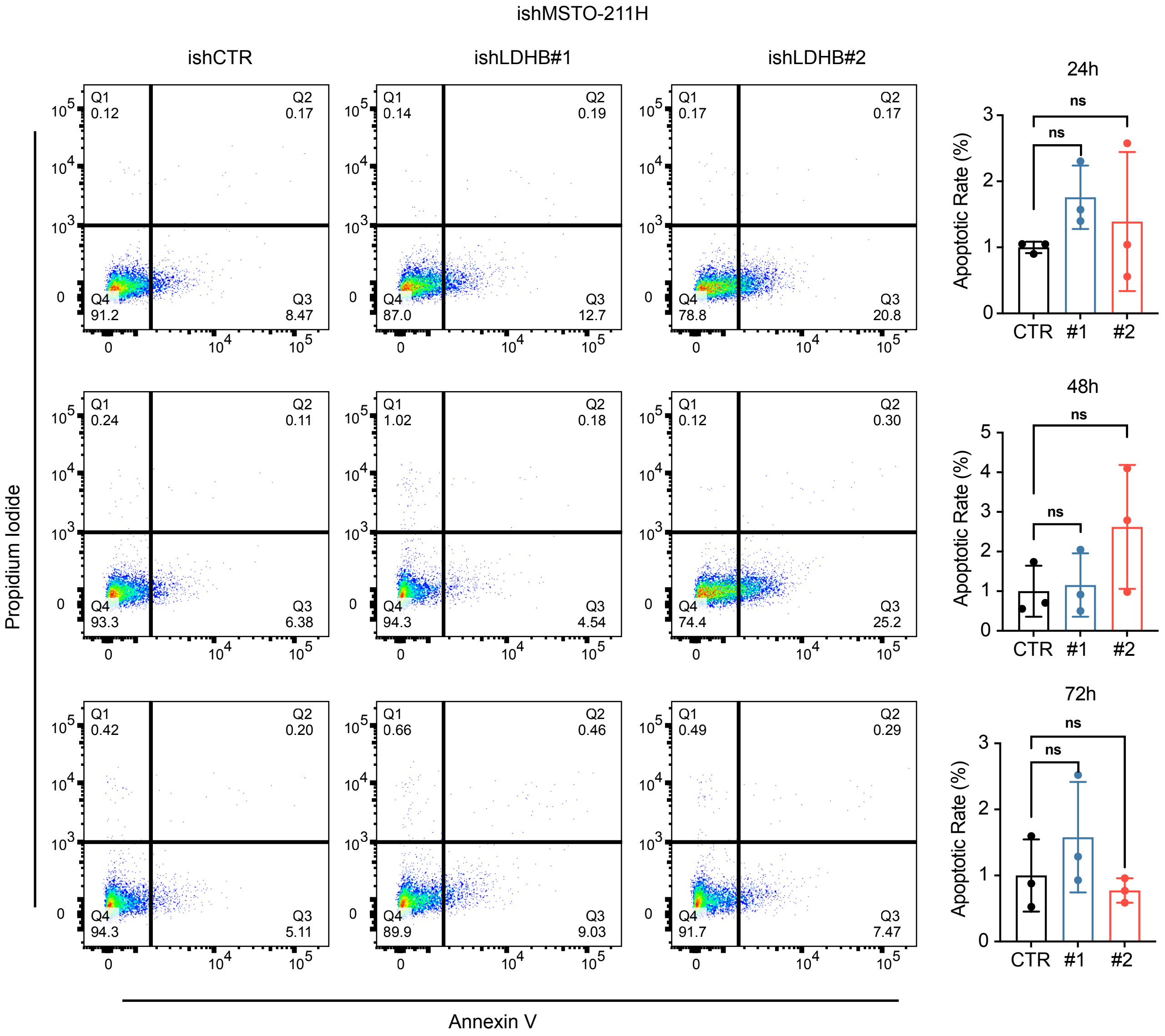
ishMSTO-211H
ishCTR
ishLDHB#1
ishLDHB#2
Propidium Iodide
Annexin V
Q1 0.12
Q2 0.17
Q4 91.2
Q3 8.47
Q1 0.14
Q2 0.19
Q4 87.0
Q3 12.7
Q1 0.17
Q2 0.17
Q4 78.8
Q3 20.8
Q1 0.24
Q2 0.11
Q4 93.3
Q3 6.38
Q1 1.02
Q2 0.18
Q4 94.3
Q3 4.54
Q1 0.12
Q2 0.30
Q4 74.4
Q3 25.2
Q1 0.42
Q2 0.20
Q4 94.3
Q3 5.11
Q1 0.66
Q2 0.46
Q4 89.9
Q3 9.03
Q1 0.49
Q2 0.29
Q4 91.7
Q3 7.47
24h
48h
72h
Apoptotic Rate (%)
ns
CTR
#1
#2

Figure S5. LDHB inhibition does not induce apoptosis in MSTO-211H cells. Flow cytometry analysis of apoptosis in ishMSTO-211H cells (left panel) with different time point. Quantification of apoptotic rate in corresponding time point was normalized to ishCTR (n=3) (right panel). Cells were treated with 0.5µg/mL doxycycline for 24-72 hours to induce shLDHB effect. All data represent means ± SD. *$p<0.05$, **$p<0.01$, ***$p<0.001$, ****$p<0.0001$; ns, not significant; by Student's t test, unpaired.

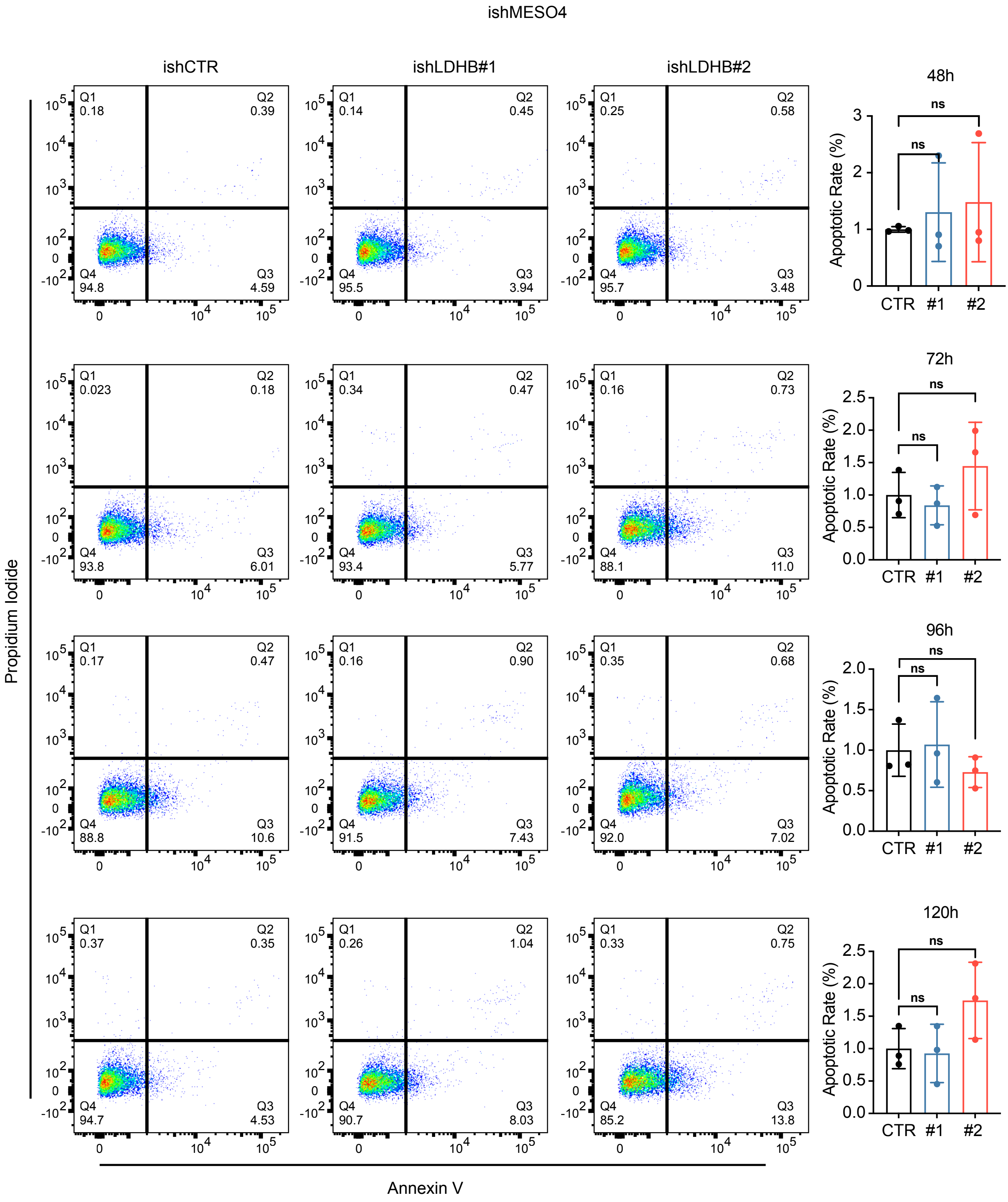
ishMESO4
ishCTR
ishLDHB#1
ishLDHB#2
Propidium Iodide
Annexin V
48h
72h
96h
120h
Apoptotic Rate (%)
ns
CTR
#1
#2
Q1 0.18
Q2 0.39
Q4 94.8
Q3 4.59
Q1 0.14
Q2 0.45
Q4 95.5
Q3 3.94
Q1 0.25
Q2 0.58
Q4 95.7
Q3 3.48
Q1 0.023
Q2 0.18
Q4 93.8
Q3 6.01
Q1 0.34
Q2 0.47
Q4 93.4
Q3 5.77
Q1 0.16
Q2 0.73
Q4 88.1
Q3 11.0
Q1 0.17
Q2 0.47
Q4 88.8
Q3 10.6
Q1 0.16
Q2 0.90
Q4 91.5
Q3 7.43
Q1 0.35
Q2 0.68
Q4 92.0
Q3 7.02
Q1 0.37
Q2 0.35
Q4 94.7
Q3 4.53
Q1 0.26
Q2 1.04
Q4 90.7
Q3 8.03
Q1 0.33
Q2 0.75
Q4 85.2
Q3 13.8

Figure S6. LDHB inhibition does not induce apoptosis in MESO4 cells. Flow cytometry analysis of apoptosis in ishMESO4 cells (left panel) with different time point. Quantification of apoptotic rate in corresponding time point was normalized to ishCTR (n=3) (right panel). Cells were treated with 0.05µg/mL doxycycline for 48-120 hours to induce shLDHB effect. All data represent means ± SD. *$p<0.05$, **$p<0.01$, ***$p<0.001$, ****$p<0.0001$; ns, not significant; by Student's t test, unpaired.

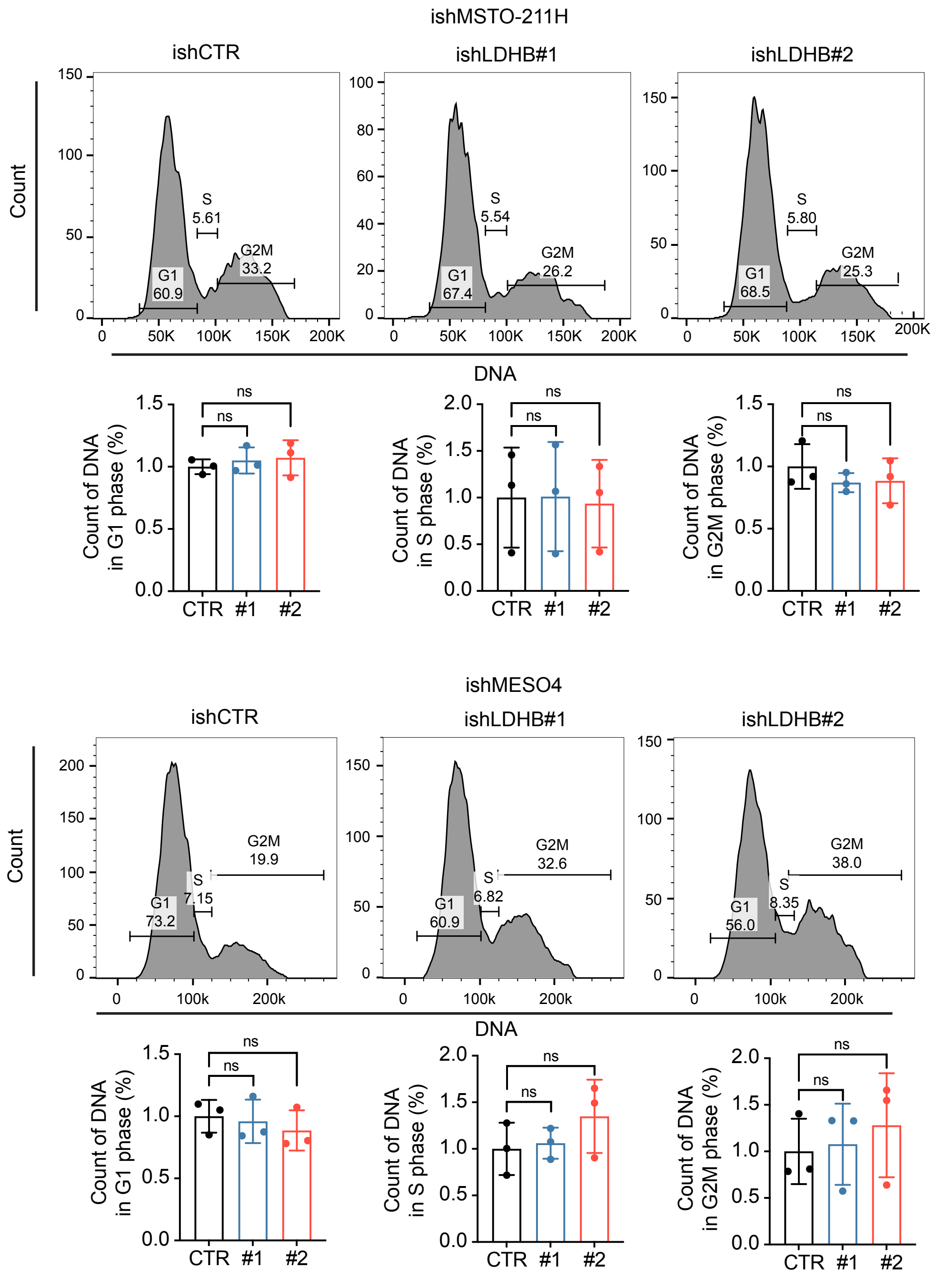
ishMSTO-211H
ishCTR
ishLDHB#1
ishLDHB#2
Count
S 5.61
G1 60.9
G2M 33.2
S 5.54
G1 67.4
G2M 26.2
S 5.80
G1 68.5
G2M 25.3
DNA
Count of DNA in G1 phase (%)
Count of DNA in S phase (%)
Count of DNA in G2M phase (%)
ns
CTR #1 #2
ishMESO4
ishCTR
ishLDHB#1
ishLDHB#2
Count
G2M 19.9
S 7.15
G1 73.2
G2M 32.6
S 6.82
G1 60.9
G2M 38.0
S 8.35
G1 56.0
DNA
Count of DNA in G1 phase (%)
Count of DNA in S phase (%)
Count of DNA in G2M phase (%)
ns
CTR #1 #2

Figure S7. LDHB inhibition does not induce cell cycle arrest in PM cells. Flow cytometry analysis of cell cycle in ishMSTO-211H and ishMESO4 cells by DAPI. Cells were treated with 0.5μg/mL and 0.05μg/mL doxycycline for 72 hours and 120 hours respectively. Quantification in corresponding cell cycle phase was normalized to ishCTR (n=3). All data represent means ± SD. *p<0.05, **p<0.01, ***p<0.001, ****p<0.0001; ns, not significant; by Student's t test, unpaired.

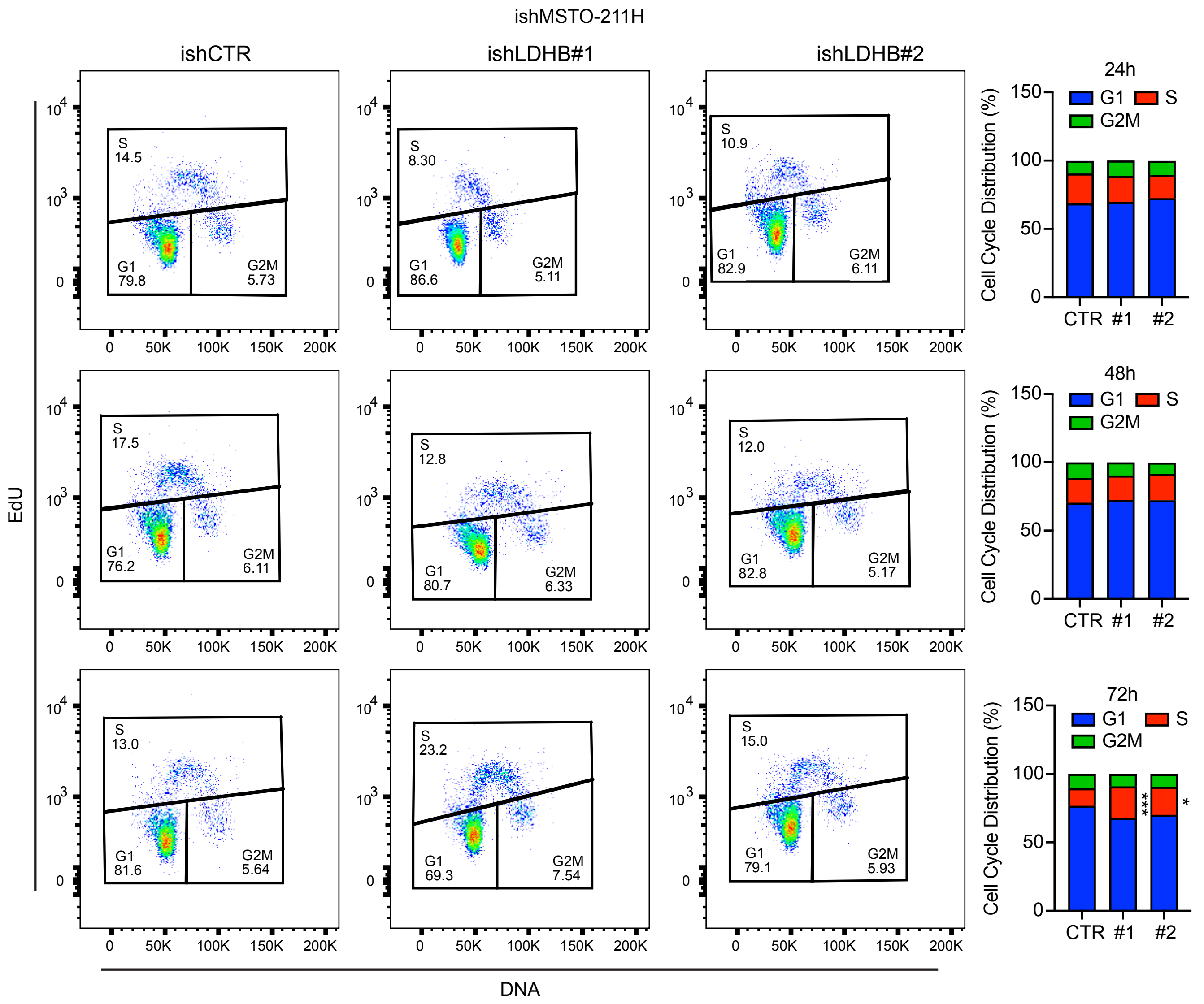

ishMSTO-211H
ishCTR
ishLDHB#1
ishLDHB#2
EdU
DNA
S 14.5
G1 79.8
G2M 5.73
S 8.30
G1 86.6
G2M 5.11
S 10.9
G1 82.9
G2M 6.11
S 17.5
G1 76.2
G2M 6.11
S 12.8
G1 80.7
G2M 6.33
S 12.0
G1 82.8
G2M 5.17
S 13.0
G1 81.6
G2M 5.64
S 23.2
G1 69.3
G2M 7.54
S 15.0
G1 79.1
G2M 5.93
24h
48h
72h
G1 S G2M
Cell Cycle Distribution (%)
CTR #1 #2
***
*

Figure S8. LDHB inhibition induces cell cycle arrest in MSTO-211H cells. Flow cytometry analysis of EdU incorporation in ishMSTO-211H cells (left panel) with different time point. Quantification of EdU in corresponding cell cycle phase (n=3) (right panel). Cells were treated with 0.5µg/mL doxycycline for 24-72 hours to induce shLDHB effect. All data represent means ± SD. *$p<0.05$, **$p<0.01$, ***$p<0.001$, ****$p<0.0001$; ns, not significant; by Student's t test, unpaired.

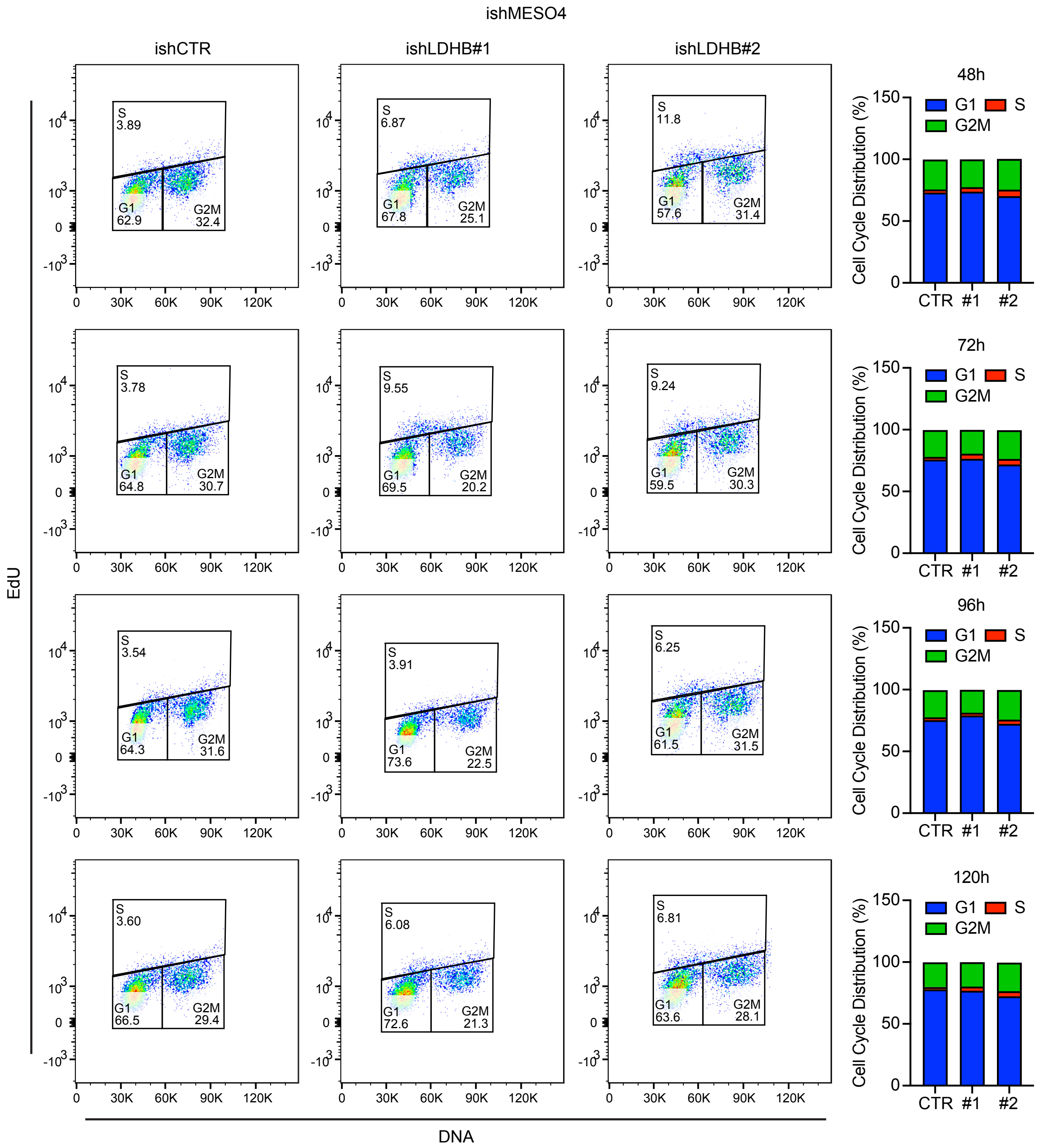
ishMESO4
ishCTR
ishLDHB#1
ishLDHB#2
EdU
DNA
S 3.89
G1 62.9
G2M 32.4
S 6.87
G1 67.8
G2M 25.1
S 11.8
G1 57.6
G2M 31.4
S 3.78
G1 64.8
G2M 30.7
S 9.55
G1 69.5
G2M 20.2
S 9.24
G1 59.5
G2M 30.3
S 3.54
G1 64.3
G2M 31.6
S 3.91
G1 73.6
G2M 22.5
S 6.25
G1 61.5
G2M 31.5
S 3.60
G1 66.5
G2M 29.4
S 6.08
G1 72.6
G2M 21.3
S 6.81
G1 63.6
G2M 28.1
48h
72h
96h
120h
Cell Cycle Distribution (%)
G1 S G2M
CTR #1 #2

Figure S9. LDHB inhibition doesn't induce cell cycle arrest in MESO4 cells. Flow cytometry analysis of Edu incorporation in ishMESO4 cells (left panel) with different time point. Quantification of Edu in corresponding cell cycle phase (n=3) (right panel). Cells were treated with 0.05µg/mL doxycycline for 48-120 hours to induce shLDHB effect. All data represent means ± SD. *$p<0.05$, **$p<0.01$, ***$p<0.001$, ****$p<0.0001$; ns, not significant; by Student's t test, unpaired.

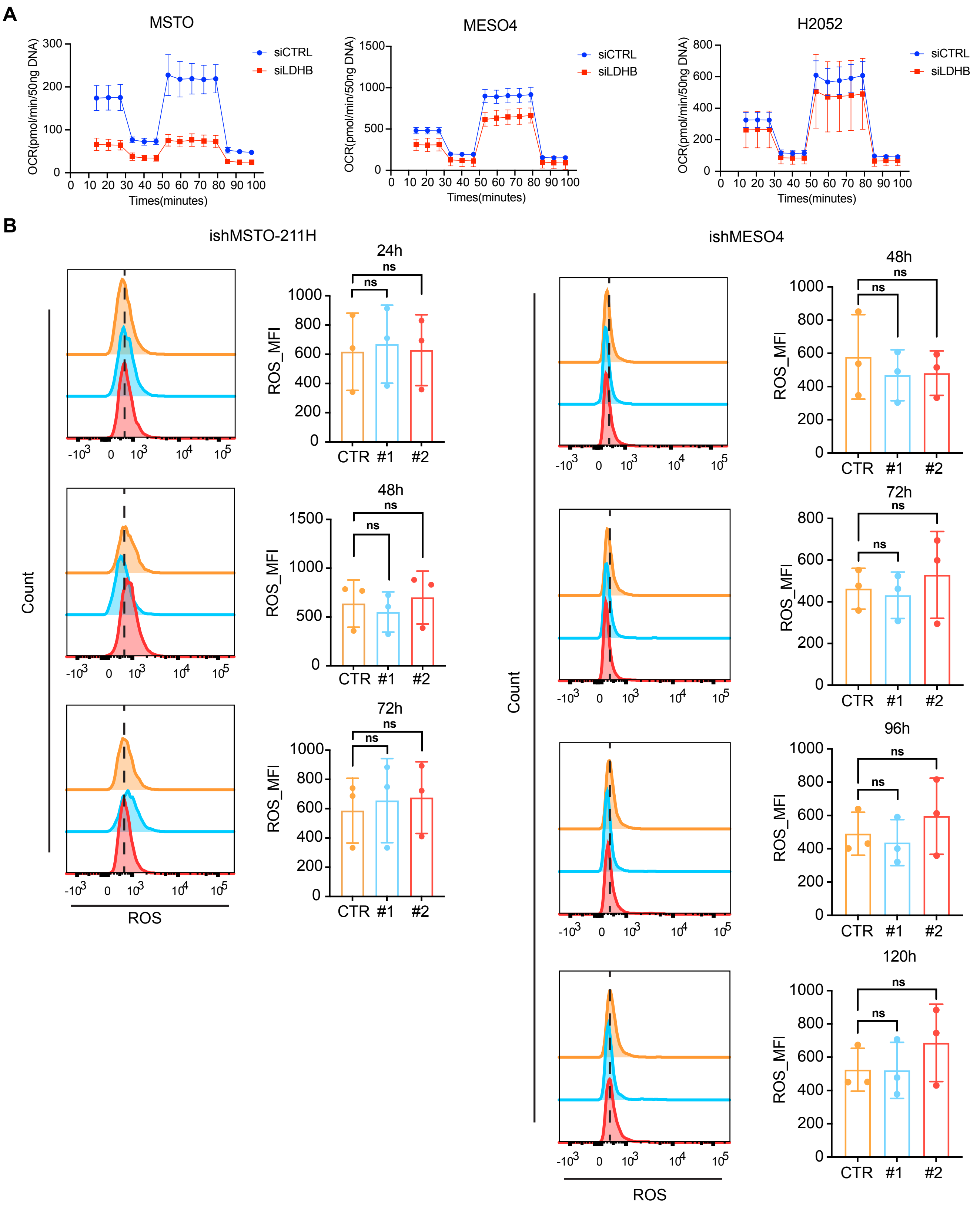
A
MSTO
MESO4
H2052
siCTRL
siLDHB
OCR(pmol/min/50ng DNA)
Times(minutes)
B
ishMSTO-211H
ishMESO4
24h
48h
72h
96h
120h
ns
ROS_MFI
CTR
#1
#2
Count
ROS

Figure S10. LDHB inhibition doesn't induce increasing ROS in MESO4 cells. A. Seahorse analysis of the real-time oxygen consumption rate (OCR) in MSTO-211H, MESO4 and H2052 siCTR and siLDHB cells (n=3). B. Flow cytometry analysis of ROS in ishMSTO-211H and ishMESO4 cells with different time point. Quantification of ROS in corresponding time point (n=3). Cells were treated with 0.5μg/mL and 0.05μg/mL doxycycline for 24-72 hours and 48-120 hours respectively. All data represent means ± SD. *p<0.05, **p<0.01, ***p<0.001, ****p<0.0001; ns, not significant; by Student's t test, unpaired.

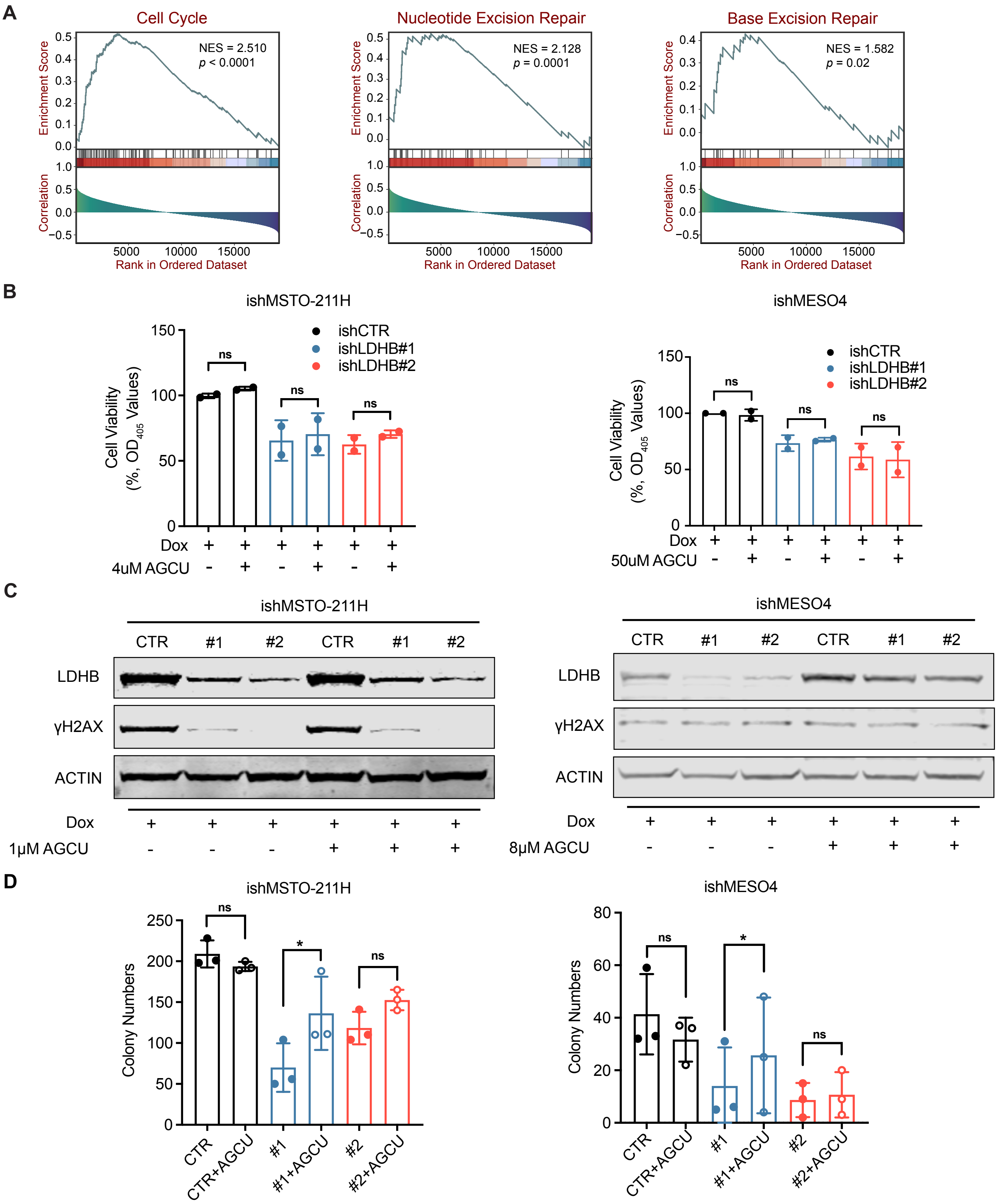
A
Cell Cycle
NES = 2.510
p < 0.0001
Nucleotide Excision Repair
NES = 2.128
p = 0.0001
Base Excision Repair
NES = 1.582
p = 0.02
Enrichment Score
Correlation
Rank in Ordered Dataset
B
ishMSTO-211H
ishMESO4
ishCTR
ishLDHB#1
ishLDHB#2
Cell Viability (%, OD405 Values)
ns
Dox
4uM AGCU
50uM AGCU
C
ishMSTO-211H
ishMESO4
CTR #1 #2 CTR #1 #2
LDHB
γH2AX
ACTIN
Dox
1μM AGCU
8μM AGCU
D
ishMSTO-211H
ishMESO4
Colony Numbers
CTR
CTR+AGCU
#1
#1+AGCU
#2
#2+AGCU
ns
*

Figure S11. LDHB correlates DNA damage response pathway in PM. A. GSEA of the TCGA dataset revealed enrichment of cell cycle, nucleotide excision repair and base excision repair pathway in KEGG for samples stratified by LDHB expression. B. Cell viability assay by acid phosphatase (APH) in ishMSTO-211H and ishMESO4 cells, treated with 0.5µg/mL and 0.05µg/mL doxycycline supplemented with or without 4µM and 50µM AGCU (adenine, guanine, cytosine and uridine) for 72h and 120h respectively, normalized to ishCTR (n=2). C. Western blot analysis of LDHB and γH2AX expression in ishMSTO-211H and ishMESO4 cells. Cells were treated with 0.5µg/mL and 0.05µg/mL doxycycline supplemented with or without 1µM and 8µM AGCU (adenine, guanine, cytosine and uridine) for 72h and 120h respectively (n=3). D. Quantification of colony numbers in ishMSTO-211H and ishMESO4 cells (n=3). All data represent means ± SD. *$p<0.05$, **$p<0.01$, ***$p<0.001$, ****$p<0.0001$; ns, not significant; by Student's t test, unpaired.

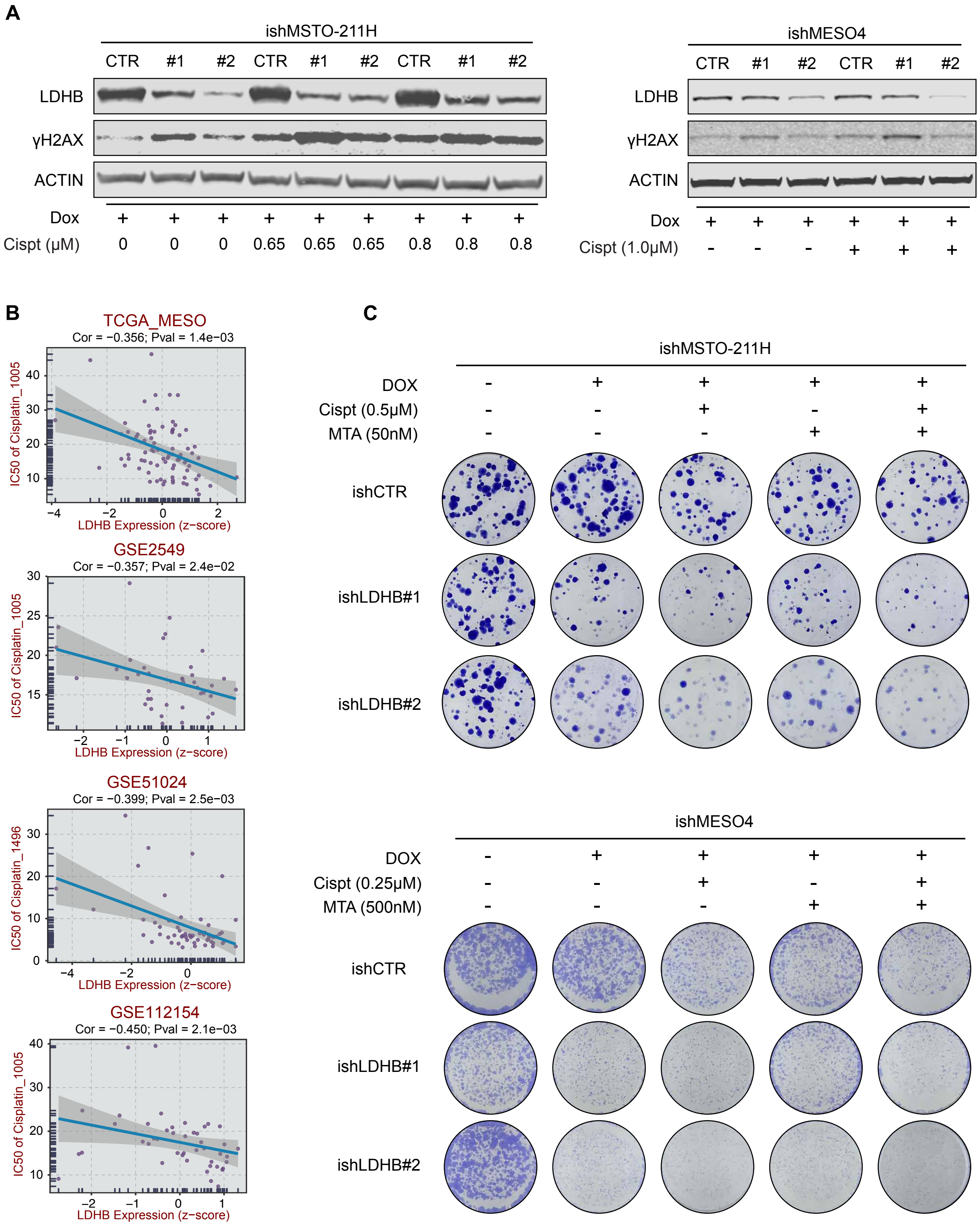

A
ishMSTO-211H
CTR #1 #2 CTR #1 #2 CTR #1 #2
LDHB
γH2AX
ACTIN
Dox + + + + + + + + +
Cispt (μM) 0 0 0 0.65 0.65 0.65 0.8 0.8 0.8
ishMESO4
CTR #1 #2 CTR #1 #2
LDHB
γH2AX
ACTIN
Dox + + + + + +
Cispt (1.0μM) - - - + + +
B
TCGA_MESO
Cor = −0.356; Pval = 1.4e−03
IC50 of Cisplatin_1005
LDHB Expression (z−score)
GSE2549
Cor = −0.357; Pval = 2.4e−02
IC50 of Cisplatin_1005
LDHB Expression (z−score)
GSE51024
Cor = −0.399; Pval = 2.5e−03
IC50 of Cisplatin_1496
LDHB Expression (z−score)
GSE112154
Cor = −0.450; Pval = 2.1e−03
IC50 of Cisplatin_1005
LDHB Expression (z−score)
C
ishMSTO-211H
DOX - + + + +
Cispt (0.5μM) - - + - +
MTA (50nM) - - - + +
ishCTR
ishLDHB#1
ishLDHB#2
ishMESO4
DOX - + + + +
Cispt (0.25μM) - - + - +
MTA (500nM) - - - + +
ishCTR
ishLDHB#1
ishLDHB#2

Figure S12. LDHB silencing sensitize PM cells to cisplatin treatment. A. Western blot analysis of LDHB and γH2AX expression in ishMSTO-211H and ishMESO4. Cells were treated with doxycycline alone as described before or in combination with various concentration of cisplatin 24h after seeding for 72h and 120h, quantification of the expression level was normalized to β-actin (ACTIN) (n=3). B. Correlation of LDHB expression with cisplatin sensitivity of PM in TCGA and GEO dataset. C. Colony formation assay in ishMSTO-211H and ishMESO4 cells. Cells were treated with normal culture medium, medium with 0.5μg/mL and 0.05μg/mL doxycycline alone or in combination with cisplatin or pemetrexed (MTA), respectively, for 7-15days (n=3). All data represent means ± SD. $^*p<0.05$, $^{**}p<0.01$, $^{***}p<0.001$, $^{****}p<0.0001$; ns, not significant; by Student's t test, unpaired.

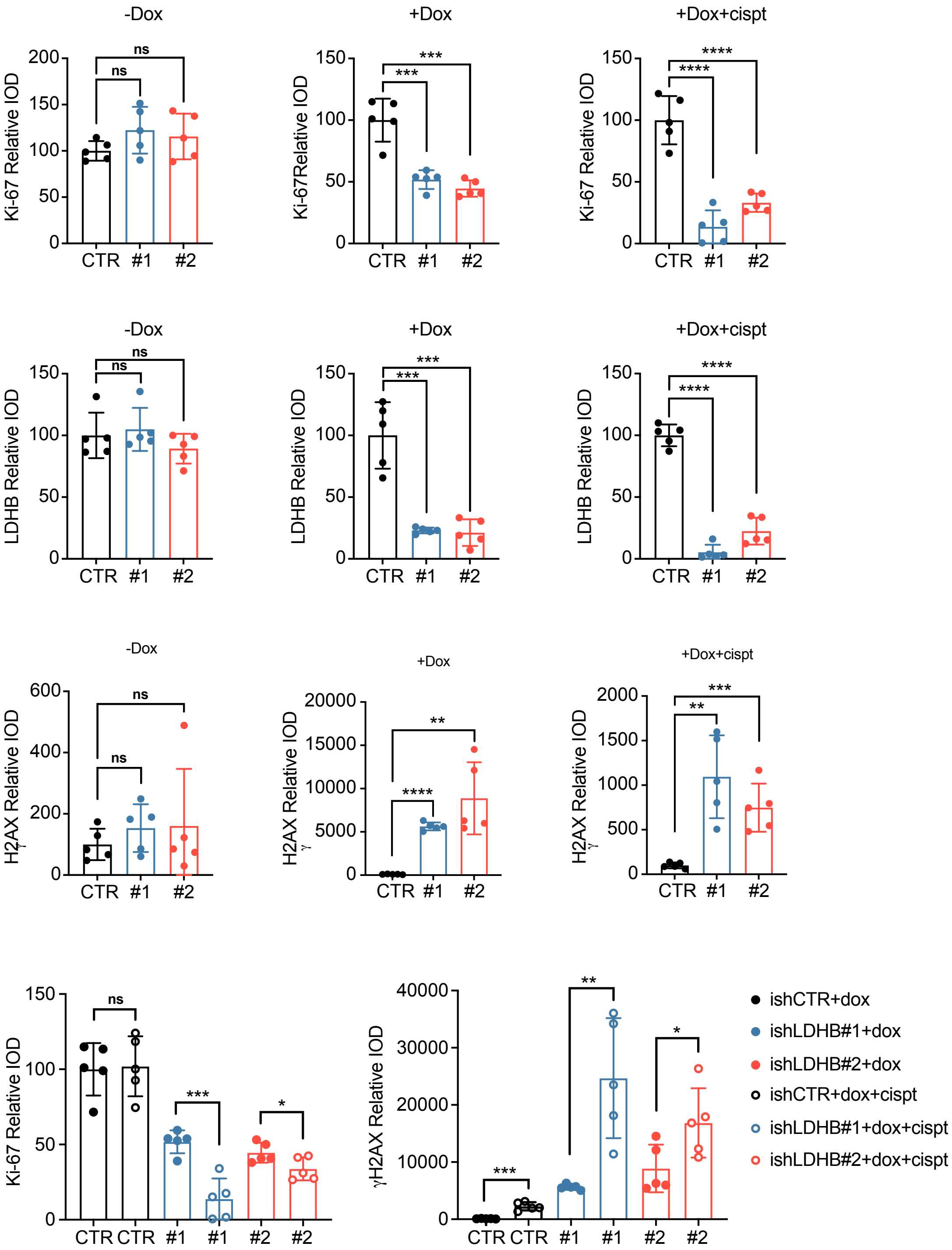
-Dox
+Dox
+Dox+cispt
Ki-67 Relative IOD
Ki-67Relative IOD
LDHB Relative IOD
γH2AX Relative IOD
CTR
#1
#2
ns
*
**
***
****
ishCTR+dox
ishLDHB#1+dox
ishLDHB#2+dox
ishCTR+dox+cispt
ishLDHB#1+dox+cispt
ishLDHB#2+dox+cispt

Figure S13. LDHB silencing sensitize PM cells to cisplatin treatment in vivo. Quantification of IHC analysis for Ki-67, LDHB and γH2AX in ishMSTO-211H xenograft tumors. All data represent means ± SD. *p<0.05, **p<0.01, ***p<0.001, ****p<0.0001; ns, not significant; by Student's t test, unpaired.

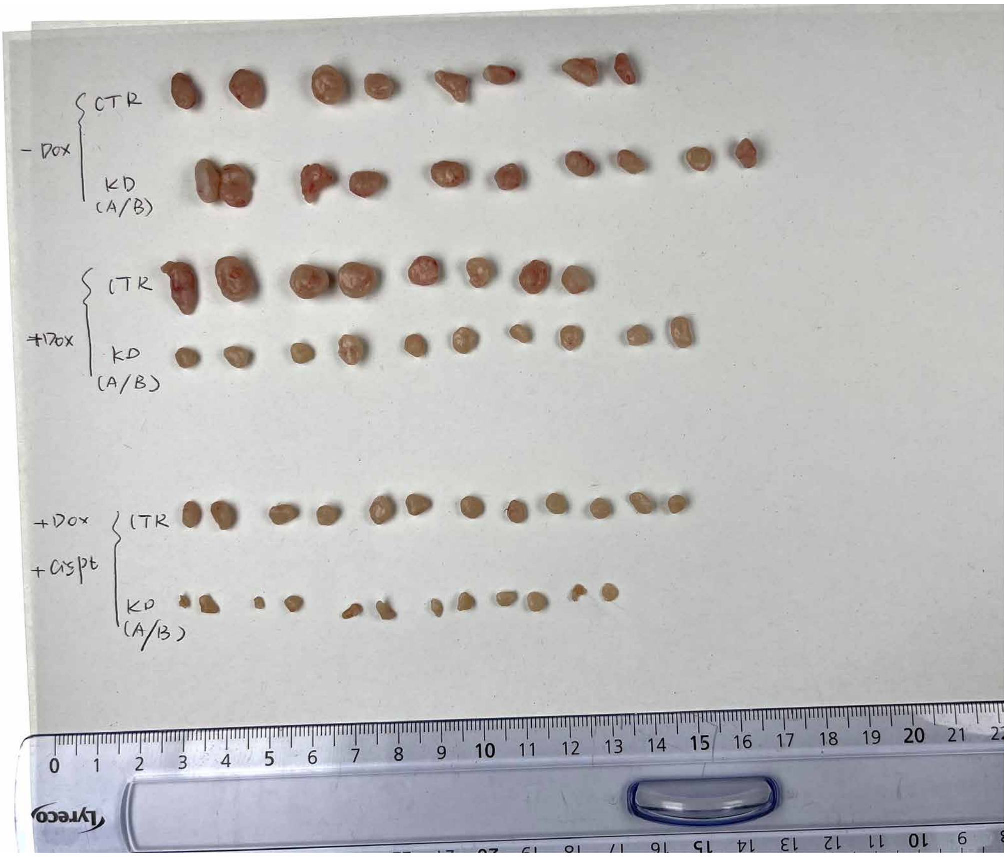

CTR
- Dox
KD (A/B)
CTR
+Dox
KD (A/B)
+Dox
+Cispt
CTR
KD (A/B)
Lyreco

Figure S14. LDHB silencing sensitize PM cells to cisplatin treatment in vivo. Related to Fig. 5B: Image of ishMSTO-211H xenograft tumors, including a ruler for scale.
